# Effect of Variety and Maturity Index on the Physicochemical Parameters Related to Virgin Olive Oil from Wudu (China)

**DOI:** 10.3390/foods12010007

**Published:** 2022-12-20

**Authors:** Fengxia Tang, Chuan Li, Xiaoran Yang, Jiandu Lei, Hongxia Chen, Changwei Zhang, Chengzhang Wang

**Affiliations:** 1 Institute of Chemical Industry of Forest Products, Chinese Academy of Forestry, Nanjing 210042, China; 2 College of Materials Science and Technology, Beijing Forestry University, Beijing 100083, China; 3 Co-Innovation Center of Efficient Processing and Utilization of Forest Resources, Nanjing Forestry University, Nanjing 210042, China

**Keywords:** virgin olive oil, chemical compounds, maturity index, variety, physical parameters

## Abstract

Physical parameters (i.e., extraction yield, oil content), chemicals (i.e., fatty acids, phenolics) and oxidative stability associated with virgin olive oil (VOO) from ten varieties in Wudu, China, were analyzed as a function of maturity index and variety by multivariate analysis models. Most of the analytical parameters were significantly affected by the variety and maturity index, and the former was more influential than the latter. Phenolics were the principal factor dividing the ten varieties into four groups. High phenolic levels were observed in the ‘*Koroneiki*’ group and ‘*Manzanilla*’ group, but the oil extractability index differentiated between them, being the highest and lowest, respectively. The ‘*Koroneiki*’ group demonstrated high oil productivity and oil quality, which was worthy of promotion in large-scale cultivation. High amounts of linoleic enhanced the VOO health benefits of ‘*Ascolana tenera*, *Arbequina* and *Zhongshan24*’ group, but brought the risk of shortening the shelf-life. The ‘*Ulliri Bardhe*, *Empeltre*, *Ezhi8*, *Yuntai14* and *Picual*’ group clustered for the higher relative value of oleic acid. The maturity index had significant negative effects on the content of total phenolics, oleacein, oleocanthal, and oleic acid, but had a positive effect on the extractability index, which suggested that varieties with low phenolics and oleic acid levels should be harvested early.

## 1. Introduction

Olea europaea (*Olea europaea* L.) is a traditional agroforestry crop in the Mediterranean that generates considerable commercial value of seven billion euros every year and has become an important economic pillar in the Mediterranean. Virgin olive oil (VOO) is one of the most important derivative products [1]. Olives have been grown in China for over 60 years and Wudu has first-class suitability as an area for olive cultivation, but only 21 of the 165 introduced olive varieties (for oil production) are perfectly adaptable to the local environment [2]. Generally, the well-adapted varieties in Wudu were primarily screened out based on physical indicators (e.g., fruit production, oil content), with little relationship to VOO chemical composition [3]. In recent years, the chemical components in VOO have been gradually reported, and mostly focus on the composition of fatty acids (FAs), volatile compounds and simple phenols [4,5,6]. However, there are limited reports on secoiridoids, which are recognized as important organoleptic compounds in VOO. This is detrimental to controlling sensorial characteristics and the quality of VOO. Varietal selection criteria in olive practice should be comprehensively considered.

Several physical indicators related to olive fruit (e.g., oil content, moisture, abencor yield (AY) and extractability index (EI)) were suggested to assess oil productivity [7]. FA composition and phenolics have been the primary chemicals to assess the VOO quality and oxidation stability, thanks to their excellent nutrition and antioxidant activities protecting VOO from thermo-oxidizing and self-oxidation [8,9]. Factors affecting the above physicochemical parameters can be categorized into the following five categories: process technology (i.e., extraction conditions, centrifugation) [10], harvesting (i.e., maturity index (MI)) [11], meteorology (i.e., rainfall, temperature) [12], genotype (i.e., variety) [11], and agronomy (i.e., irrigation regimes, fertilizer) [13]. The effect of variety and MI on VOO characteristics were reported to assess the adaptability of olive varieties to the local environment for the local agricultural department.

Generally, oil accumulation continues until the fruits fully mature, but oil extraction yield decreases or increases with the maturity degree of olive fruits [14]. Variations in oil content and extraction yield determine the oil extractable quantity. Oleic acid (16:1) is the highest fatty acid in VOO, which ranges from 55% to 83% according to the varieties and MI. The secondary fatty acids in VOO are palmitic acid (16:0) and linoleic (18:2), which are 7.5–20% and 3.50–21.00%, respectively. FAs composition constantly changes as the activity of oleate desaturase changes with the growth of the olives [15]. Other FAs, such as docosanoic acid, are non-negligible for monitoring VOO quality and vary significantly between cultivars [16]. Secoiridoids, representative phenolics in VOO, are responsible for organoleptic characteristics, health benefits, and oxidation stability [17]. Aglycone of oleuropein and ligstroside, oleocanthal, and oleacein are the major secoiridoids and exhibit cultivar-specific traits [18,19]. Other lower concentrations of phenolic compounds, such as hydroxytyrosol, also have important effects on the oil quality [9]. In general, the concentrations of total phenolics are negatively correlated with the olive growth process in most varieties, contrary to oil accumulation in the fruit [20]. The choice of the optimal harvesting time should not compromise the maximum quantity and quality of VOO.

Different expression levels of physicochemical parameters of the same variety were ascribed to differences in planting conditions [21]. As the birthplace of olive cultivation, the weather in the Mediterranean is characterized by hot, dry summers and warm, wet winters, while Wudu has hot, humid summers and cold, dry winters. Quantitative measurements of physicochemical parameters help extend the currently limited knowledge concerning varietal characteristics under non-Mediterranean climates. Tracking variations in VOO characteristics provides information for harvesting. In view of the importance of varietal selection and harvesting for oil production, physicochemical characteristics of VOO from ten common olive cultivars were analyzed with three maturity indices (MI: 2, 3, 4) in Wudu, China, and the information on the selected varieties is shown in Table 1. Additionally, clone *Zhongshan24*, was obtained by large-scale selection of free-pollinated seedlings of the cultivar *Ascolana Tenera*. Statistical methods were applied in the multivariate analysis of measurement.

## 2. Materials and Methods

### 2.1. Chemical Reagents

FA methyl ester mixture (≥98%) was purchased from Sigma-Aldrich (Shanghai, China). Folin Ciocalteu phenol reagent, petroleum ether, sodium carbonate, and sodium hydroxide were of analytical grade and gained from Sinopharm Medicine Holding Co., Ltd. (Shanghai, China). Hydroxytyrosol, tyrosol, vanillin, vanillic acid, caffeic acid, p-coumaric acid, pinoresinol, luteolin, apigenin, acetic acid and gallic acid were 98% and obtained from Aladdin Co., Ltd. (Shanghai, China). Methanol and acetonitrile for HPLC were of chromatographic grade and obtained from Tedia Co., Ltd. (Shanghai, China). Oleacein (≥98%), oleocanthal (≥98%), monoaldehydic form of oleuropein aglycone (≥90%), monoaldehydic form of ligstroside aglycone (≥90%) were provided by Maria′s research group (National and Kapodistrian University of Athens, Greece).

### 2.2. Area of Study

Samples were collected in 2021 at the Dabao Olive Variety Experimental Park of Wudu Economic Forestry Research Institute, located in Wudu, Gansu (33°24′03″ N, 104°53′30″ E, altitude of 1036–1048 m), southwest China. Wudu has a subtropical monsoon climate with annual mean, minimum and maximum temperatures of 15.3 °C, −1 °C (January), and 31 °C (July), respectively. Precipitation is concentrated between June and September, with an annual average of 468 mm. The relative humidity is 56.6% and the sunshine time is 1871 h. The soil is sandy loam with a pH of 7.9 [22] (http://data.cma.cn/site/index.html, accessed on 5 December 2022).

### 2.3. Olive Collection and Analysis

In olive practices, it is convenient to judge the maturity state of fruit based on the color of the peel and flesh, which are generally divided into seven maturity indices [23]. Two trees were selected for each variety and 6 kg were picked manually at three maturity indices (MI: 2, 3, 4), respectively. Three batches were sampled independently in three separate areas. Determination of dry matter oil content (DMO), moisture content (MC), and wet matter oil content (WMO) of olive were described previously [24].

### 2.4. Abencor Yield and Extractability Index of VOO Extraction

The oil extraction yield was accessed by the Abencor extraction system (MC2 Ingeniería y Sistemas, Sevilla, Spain) in the laboratory, but this could not fully reflect industrial production in reality. VOO extraction was conducted in accordance with the method described by Polari et al. and modified [24]. The pastes were stirred at 28 °C for 30 min, and then centrifuged at 3000 rpm for 1 min twice. The upper oil phase was filtered and stored in brown bottles at −20 °C until analysis. Abencor yield (AY, %), representing the industrial yield, was calculated by the following equation [25]: AY= 100% × V×ρ/m, where V (ml) was the volume of extracted VOO, ρ was the density of VOO (0.915 g/cm^3^), and m (g) is the weight of the olive paste.

The extractability index (EI, %), a comprehensive indicator, was calculated using the following formula [25]: EI = 100% × AY/W, Where W (%) was the oil content of wet matter, and AY (%) was abencor yield.

### 2.5. VOO Sample Analyses

#### 2.5.1. FAs Composition

The quantitation method of FAs was performed as Yan et al. reported [22]. FAs, after being converted into methyl esters, were analyzed by Gas Chromatography (Thermo Fisher Trace 1300 ISQ, Thermo Fisher Scientific, MA, USA) coupled with a Triple Quadrupole Mass Spectrometry (GC-MS) (Thermo Fisher Trace 1300 ISQ, Thermo Fisher Scientific, MA, USA), and equipped with AE-FFAP capillary column (30 m × 0.25 mm × 0.25 µm). Purified helium (>99.99%) was used as a carrier gas with a flow rate of 1 mL/min. The injection volume was 1 µL with a split ratio of 10. As for gas chromatographic conditions, the initial oven temperature was maintained at 70 °C for 4 min, and then increased to 190 °C at 10 °C/min for 8 min, followed by a gradient of 4 °C/min to a final temperature of 250 °C for 5 min. The mass spectrometric condition was conducted in electron ionization mode with collision energy at 70 Ev. The transmission line temperature and the ion source temperature were 250 °C and 280 °C, respectively. The mass spectra were set to full-scan mode with a mass scan range of *m*/*z* 50–800 and the solvent delay was set to 2 min. The retention time of each FA standard was checked with the mass spectral database (National Institute of Standards and Technology (NITS) 2011, and the relative contents of the main FAs in VOO were calculated based on the peak area normalization method, expressed as percentages of total FAs (%).

#### 2.5.2. Phenolic Compositions

A quantity of 3 g VOO was extracted with 5 mL of methanol/water (80:20, *v*/*v*) in a 30 °C water bath, and stirred at 250 rpm for 30 min under nitrogen condition. The supernatant was collected and dried under the vacuum atmosphere of 60 °C, and then dissolved in 1 mL of methanol for further detection. Quantification of phenolics was performed by HPLC equipped with a diode array detector (Shimadzu Prominence LC-20A, Shimadzu, Kyoto, Japan) and a Nuclosil 100-5 C18 column (250 × 4.6 mm, 5 µm, Macherey-Nagel, Nordrhein-Westfalen, Germany). The chromatographic conditions were modified according to Ricciutelli et al.’s method [26]. The mobile phase was 0.2% acetic acid aqueous solution (A) and acetonitrile/methanol (1:1, *v*/*v*) (B). The flow rate was 1 mL/min and the detection wavelength was 280 nm. The gradient elution procedure was at 0–40 min: 4–50% B, 40–50 min: 50–40% B, 50–60 min: 100% B, 60–70 min: 100–4% B, 70–82 min: 4% B. Phenolic compounds were identified by Thermo Scientific mass spectrometer (LTQ-Orbitrap XL, Thermo Fisher Scientific, MA, USA). The mass spectrometry was conducted in negative ion mode and the ESI source setting conditions were as follows: source heater temperature, 300 °C; sheath gas flow rate, 700 L/h; cone gas flow rate, 50 L/h; capillary temperature, 320 °C; capillary voltage, −10 V; ion spray voltage, 2.9 kV. The retention time of the main phenolic compounds is summarized in Table S1 and the MS chromatograms presented in Figure S1. The mixture of phenolics standards (tyrosol, hydroxytyrosol, vanillin, vanillic acid, caffeic acid, p-coumaric acid, elenolic acid, pinoresinol, luteolin, apigenin, oleacein, oleocanthal, monoaldehydic form of oleuropein and ligstroside aglycone) were utilized to build the calibration curve for phenolics compounds. Acetoxypinoresinol was quantified according to the pinoresinol calibration curve. The monoaldehydic form of oleuropein aglycone and monoaldehydic form of ligstroside aglycone were unified throughout the full paper as oleuropein aglycone and ligstroside aglycone.

TPP concentration was measured according to the method of Wang et al. [27]. In short, 50 µL of methanol extract was combined with 1 mL of Folin-Ciocalteu reagent (1:1) and 2.5 mL of sodium carbonate (0.15 g/mL). The mixture was shaken for 30 s with an Ultraturrax homogenizer, deionized water was added until the final volume reached 10 mL, and then reacted in the dark at room temperature for 120 min. Measurements were conducted at 750 nm with a UV spectrophotometer (UV-1800 UV-Vis, Shanghai Meipuda Instrument Co., Ltd, Shanghai, China), expressed as mg·kg−1 (equivalent amount of gallic acid).

#### 2.5.3. Oxidative Stability

Oxidative stability was performed by the Rancimat 743 apparatus (Metrohm, Shanghai, China) and expressed as induction time, h. Briefly, a 3.0 g of oil sample was heated at 120 ± 1.6 °C with an airflow rate of 20 L/h [28].

### 2.6. Statistical Analysis

All results were recorded as mean ± standard deviation in supporting materials. Due to some data not conforming to the homogeneity of variance and normal distribution, permutational multivariate analysis of variance (PERMANOVA) was applied to investigate the comprehensive effects of variety and maturity index on the studied variables. One-way ANOVA was used to obtain the significance of variety and maturity index to the variables studied, respectively. Orthogonal projection to latent structures discriminant analysis (OPLS-DA) was used to discriminate varieties and find the most important variables by VIP function. In addition, OPLS was used to explore the parameters most influenced by the maturity index by S-Plot [19]. R was responsible for the PERMANOVA, one-way ANOVA, and Spearman’s correlation analysis [29]. SIMCA-P 13.0 (America, Umetrics) was used for OPLS and OPLS-DA analysis. All data were normalized by z-core and the heat map was plotted using the online platform (http://www.bioinformatics.com.cn, accessed on 13 march 2022).

## 3. Results and Discussion

### 3.1. Maturity Index

In early September, the epidermal color of olive fruits gradually changed from green to yellow (MI: 0 to 2). From the end of September to the end of October, the skin color of most varieties changed from yellow to black-purple (MI: 2 to 4). Except for late maturing cultivars, such as *Ezhi8*, which were still between 2 and 4 by mid-November (harvesting end date), the MI of most varieties was above 4. As the temperature in Wudu rapidly dropped to below 0 °C after mid-November, frosty weather would severely affect the quality and sensory of VOO [30]. Therefore, three maturity indices (MI: 2, 3, 4) were selected for further study.

### 3.2. Physicochemical Parameters Analysis

**Figure 1:**
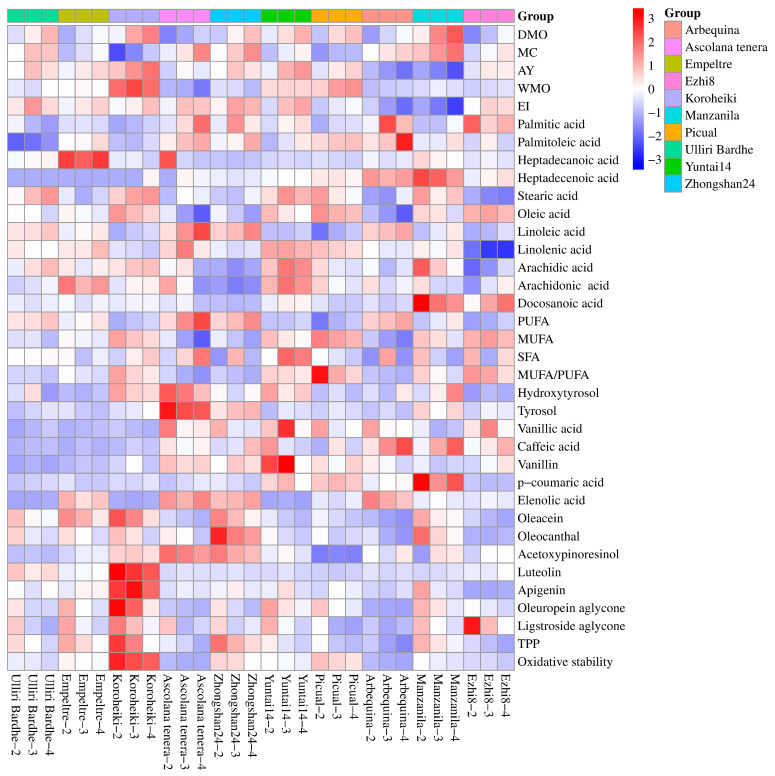
Heat-map visualizing the level of quantity and quality characteristics of VOO from ten varieties with three maturity indices (MI: 2, 3, 4). Red represents high level, and blue represents low level. ^1^Dry matter oil content (DMO); moisture content (MC); wet matter oil content (WMO); extractability index (EI); abencor yield (AY); saturated fatty acid (SFA), polyunsaturated FA (PUFA), monounsaturated fatty acid (MUFA), total polar phenolics (TPP).

A 36-variable heatmap was applied to visualize the VOO characteristic levels at harvest for ten olive varieties, with red representing high levels and blue representing low levels (Figure 1). In addition, the effect of variety, maturity index and their interaction on analytical parameters were investigated by PERMANOVA and the results presented in Table S2.

#### 3.2.1. VOO Physical Characteristics Analysis

Parameters related to oil quantity were quantified and exhibited in Figure 1 and Table S3. Oil content was assessed as DMO and WMO, and oil extraction yield was presented as AY and EI. PERMANOVA showed that the variety significantly affected all olive parameters (*p* < 0.001). The eta-squared value (*η*^2^), which explains the contribution of factors to variability, showed that more than 57.5% of variations could be attributed to the variety. In addition, MI significantly affected DMO, MC and EI (*p* < 0.001) and MC (*p* < 0.01). The value, *η*^2^, for the MI and the variety × MI interaction was less than 36.97%. Mean comparisons showed that *Koroneiki* demonstrated the highest oil content. due to having the highest DMO (42.21%) and WMO (19.59%), combined with the highest AY (12.43%) and the lowest MC (52.25%), but the highest EI (70.73%) was recorded in *Zhongshan24*. *Ascolana tenera* showed the lowest oil content. as it had the lowest DMO (33.87%) and WMO (12.65%). Whereas *Manzanilla* displayed the lowest oil extraction yield for the lowest AY (4.63%) and the lowest EI (32.11%), together with the highest MC (66.15%). Notably, the levels of DMO and WMO in China were lower than in the Mediterranean for the same cultivars, such as *Arbequina*, *Picual* and *Koroneiki*, as compared with previous work [14,31]. Cheng et al. observed similar results for other different cultivars, which they attributed to the fact that precipitation in China was concentrated in summer and autumn (the fruit period), while the Mediterranean region experienced concurrent droughts. Adequate irrigation increased moisture in the fruit, thus reducing the WMO content [32]. EI and AY recorded the larger coefficients of variation among the five parameters, which were 20.69 % and 29.70 %. respectively. EI was a comprehensive index reflecting the oil extractability of varieties, which was determined by oil content and extraction difficulty. *Zhongshan24* had a moderate DMO of 38.32% but the highest EI, of 70.73%, while Manzanilla showed a higher DMO of 42.10%, but the lowest EI of 32.11%. During the oil extraction process, we observed that *Manzanilla* formed homogeneous pastes that were difficult to separate, resulting in oil loss. Similar behaviors were reported by Beltrán et al., and they attributed differences in oil extractability to genetic factors [7].

DMO was positively correlated with MI, with an increase of 10.92–18.00 % during maturation. The WMO increased slightly in most varieties, due to the increase in olive fruit moisture, confirming previous work [11,12]. The behaviors of AY and EI were related to the type of olive cultivars throughout maturation. For precocious-ripening cultivars, AY and EI declined after MI above 3, whereas for most mid-late maturing cultivars, AY and EI increased continuously, but the increasing speed decreased. Exceptions were observed in *Manzanilla* and *Arbequina*, whose AY and EI declined at late ripening, which was related to their varietal characteristics. During ripening, *Manzanilla* had an increase of about 18.00% in DMO but a decrease of about 45.39% in EI. Logically, the increase in oil extraction difficulty was greater than the amount of oil production. *Ascolana tenera* and *Arbequina* also shared similar characteristics. Early harvesting is recommended for these cultivars. Other cultivars, such as *Yuntai14*, *Koroneiki* and *Empeltre*, held a continuous increase in EI coinciding with DMO, and are recommended for later harvesting.

#### 3.2.2. VOO Quality Characteristics Analysis

**Figure 2:**
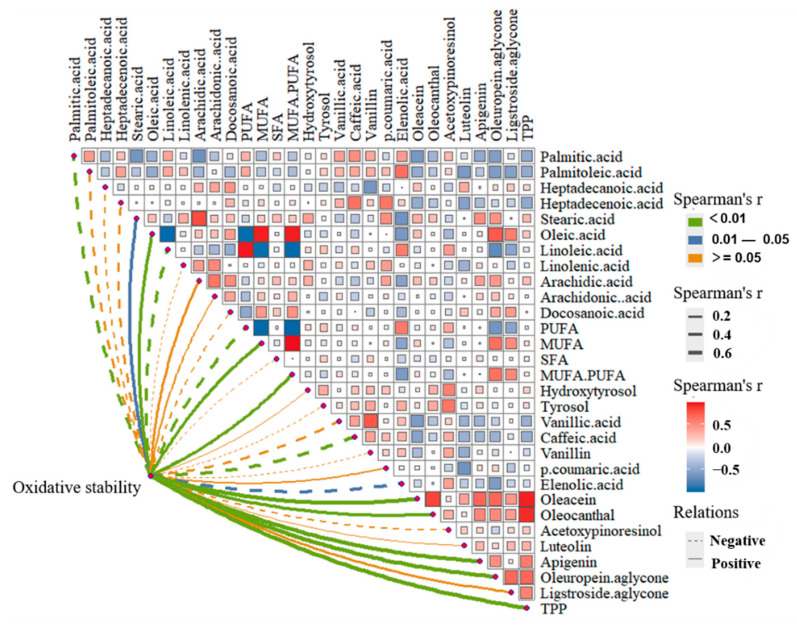
Analysis of the correlations between phenolic compounds, fatty acids and oxidative stability. ^1^Saturated fatty acid (SFA), polyunsaturated FA (PUFA), monounsaturated fatty acid (MUFA), total polar phenolics (TPP).

Spearman correlation analysis was applied to visualize the relationships between chemical parameters, and between chemical parameters and oxidative stability (Figure 2).

##### FAs Composition

FA composition, including that of eleven fatty acids, was quantified and is exhibited in Table S4. The value of each sample met the European Union requirements for extra VOOs [33]. An exception was observed in *Ascolana tenera* with more than 1% of linoleic acid (C18:3), as previously reported [11]. The IOC stated that the percentage of linoleic acid (C18:3) should not exceed 1%. FAs were classified into three groups, namely monounsaturated fatty acids (MUFAs), polyunsaturated fatty acids (PUFAs), and saturated fatty acids (SFAs). PERMANOVA indicated that the variety significantly affected all FAs (*p* < 0.001) and possessed a more prominent contribution to the variability of the FAs (*η^2^* > 60%). Moreover, MI only significantly affected oleic acid (C18:1) (*p* < 0.001), linoleic acid (C18:2) (*p* < 0.001), and palmitoleic acid (C16:1) (*p* < 0.05). The value, *η*^2^, for MI and their interaction accounted for less than 31.05%, except for the variety × MI interaction, which contributed 68.89% of the variance for SFA.

Mean comparisons showed that *Picual* was characterized by the highest percentage of MUFAs, with 73.82%, as it recorded the highest proportion of oleic acid (C18:1) of 70.09%. While *Ascolana tenera* was characterized by the highest percentage of PUFAs at 15.87%, due to the high percentages of linoleic acid (C18:2) at 14.98% and linolenic acid (C18:3) at 0.89%. In terms of SFA, *Koroneiki* exhibited the highest stearic acid (C18:0) with 4.62%, and *Ezhi8* showed the highest palmitic acid (C16:0) with 15.62%, while the highest SFA was recorded in *Yuntai14* with 19.68%. In addition, *Ascolana tenera* and its clone *Zhongshan24* had similar FA compositions, with the latter containing a slightly higher percentage of oleic acid (C18:1). The MUFA/PUFA ratio, an important indicator of oxidative stability, varied among cultivars, from 4.16 in *Ascolana tenera* to 10.11 in *Picual*. Compared with in the Mediterranean region, the FA compositions of VOO produced in Wudu exhibited parallel behavior, namely lower and higher proportions of oleic acid (C18:1) and linoleic acid (C18:2), respectively [34,35]. These results were in agreement with those of Gao et al. and Cheng et al., and were primarily due to the lower temperatures in Wudu than in the Mediterranean [12,36]. Low temperature was conducive to the synthesis of PUFAs to increase the liquidity of olive cell membranes [15]. Consequently, climate-induced changes in FA composition reduced the average MUFA/PUFA values and brought the risk of shortened shelf-life for VOO. Therefore, the same olive variety planted in the Wudu region should be harvested earlier than in Mediterranean countries, especially for varieties with lower MUFA/PUFA values, such as *Ascolana tenera*, *Arbequina* and *Zhongshan24*. During ripening, the percentages of oleic acid (C18:1) and linoleic acid(C18:2) were negatively correlated, dropping and increasing, respectively (Figure 2). These results were consistent with previous work [19], possibly due to the bioconversion of oleic acid to linoleic acid and linolenic acid in olive fruit [15]. These changes also reduced the MUFA/PUFA value and affected oil storage [37]. The rate of reduction in MUFA/PUFA values at harvesting ranged from 10.27% in *Yuntai14* to 40.69% in *Ascolana tenera*. To reduce loss of VOO shelf-life, early harvesting is recommended, mainly for varieties with rapid reduction of MUFA/PUFA value, such as *Ascolana tenera*.

##### Phenolic Composition

Fourteen phenolics in VOO from ten varieties were quantified (Table S5) and divided into the following six groups: phenolic alcohols, phenolic acids, aldehydes, flavonoids, lignans, and secoiridoids. The PERMANOVA results indicated that variety significantly impacted all phenolics (*p* < 0.001) and the variability of phenolic profiles aroused by cultivars accounted for more than 56%.

Secoiridoids were the most abundant phenolics in VOO, and their representative compounds were oleacein, oleocanthal, elenolic acid, oleuropein aglycone and ligstroside aglycone [23]. Mean comparisons showed that *Koroneiki* recorded the highest secoiridoids. as it had the highest oleacein content (212.26 mg·kg^−1^), oleuropein aglycone content (92.27 mg·kg^−1^), and ligstroside aglycone content (11.19 mg·kg^−1^). *Arbequina* held the lowest secoiridoids because it had the lowest oleacein content (41.65 mg·kg^−1^) and ligstroside aglycone content (4.71 mg·kg^−1^). Meanwhile, oleuropein aglycone and oleocanthal were also located at lower levels of 12.69 mg·kg^−1^ and 30.97 mg·kg^−1^, respectively. Oleocanthal is recognized as a natural non-steroidal anti-inflammatory drug, due to its structural similarity to ibuprofen [38]. In addition, oleocanthal is also the main compound responsible for the pungency of VOO. Apparently, *Zhongshan24* was a good source of oleocanthal, with the highest content of 127.61 mg·kg^−1^. It is worth noting that when the phenolics composition of the VOO from the two regions of Wudu and Cordoba, for the same variety (*Picual*), were compared, it was observed that the concentrations of oleuropein aglycone and ligstroside aglycone in Wudu were much lower than the concentrations of oleacein and oleocanthal [28]. Similar behaviors were also observed in the works of De Ceglie et al. and Montaño et al., which may be related to the early expression of *β*-glucosidase in the frosty climate in Wudu, because it plays a key role in shaping the phenolic composition of VOO [10,30]. A recent paper discussed the influence of frosty weather on the phenolics composition in VOO, and higher levels of oleacein were observed in frozen olive VOOs, due to the early effect of *β*-glucosidase on oleuropein in frozen olives [39]. Another observation was that elenolic acid was significantly negatively correlated with oleuropein aglycone (Figure 2). *Koroneiki* registered the highest level of oleuropein aglycone (92.27 mg·kg^−1^), and the lowest level of elenolic acid (1.16 mg·kg^−1^). *Ascolana tenera* was the variety with the highest value of elenolic acid (15.43 mg·kg^−1^) and the lowest level of oleuropein aglycone with 10.40 mg·kg^−1^. These results could be explained by the metabolic process of oleuropein in olive fruit. According to Gutierrez-Rosales et al., oleuropein hydrolysis in vivo includes two catabolism pathways, one is catalyzed by esterase and *β*-glucosidase, and eventually degraded into elenolic acid, and the other is catalyzed by *β*-glucosidase and degraded into oleuropein aglycone, which is further partially converted into oleacein [40]. Catabolism pathways of oleuropein vary from one variety to another, resulting in different phenolic profiles [41]. Low levels of ligstroside aglycones were observed in Wudu, which was likely due to the large depletion of the precursors ligstroside in the oleuropein biosynthetic pathway. Consistent with previously published works, the concentrations of secoiridoids in all cultivars showed a negative correlation with harvest, with a loss of about 32.51% to 48.89% [11]. The decline in secoiridoids is attributed to the elevated activity of endogenous enzymes in olive fruit.

With regard to lignans, the second most abundant phenolics, *Ascolana tenera* was the variety with the highest content, 26.12 mg·kg^−1^, and its improved variety *Zhongshan24* also had a higher content of 23.32 mg·kg^−1^. *Picual* only had 1.91 mg·kg^−1^. The content of lignans changed from 7.59% to 49.65% at harvest and decreased in most varieties after MI above 3, which was in agreement with previous work [19]. The coefficients of variation for phenolics ranged from 43.32% to 76.92%, with the highest and lowest being for phenolic acids, and flavonoids, respectively. *Koroneiki* had the highest flavonoid content (6.11 mg·kg^−1^), while *Ezhi8* recorded the lowest (0.54 mg·kg^−1^). As for phenolic acids, *Empeltre* held the lowest level with 2.13 mg·kg^−1^, while *Manzanilla* recorded the highest values with 7.84 mg·kg^−1^. Changes in the concentrations of flavonoids and phenolic acids varied with varieties during maturation. Notably, the phenolic acids were significantly negatively correlated with the flavonoids during the ripening process (Figure 2), which may be ascribed to their synthesis sharing a precursor compound, p-acyl-CoA [42].

Concerning TPP, *Koroneiki* registered the highest level of 447.67 mg·kg^−1^, while *Arbequina* accounted for the lowest content of 163.33 mg·kg^−1^. The concentrations of TPP in VOO were also negatively correlated with olive growth (Figure 1), due to the reduction of secoiridoid. Reduced phenolic compounds affect the quality and nutritional properties of VOO. Notably, after varietal improvement, the concentrations of total secoiridoids in *Zhongshan24* were 398.91 mg·kg^−1^, 1.81 times that of the original variety *Ascolana tenera* (220.99 mg·kg^−1^), indicating the positive effect of the breeding program on the content of phenolic compounds. *Arbequina* and *Ezhi8*, with lower contents of TPP, should be harvested earlier to preserve bioactive compounds.

##### Oxidative Stability

The values of oxidative stability of the ten varieties are summed up in Table S5. Oxidative stability decreased with the maturation process in all varieties, with *Empeltre* being the variety with the lowest loss at 12.95%, while *Zhongshan24* had the highest loss at about 25.09%. The decrease was attributed to variations in chemical composition in VOO at harvest. Figure 2 shows that oleic acid (C18:1), oleuropein aglycone, oleacein, TPP, and apigenin were highly positively correlated with oxidative stability, and all compounds showed downward trends during fruit ripening, except for apigenin. Linoleic acid (C18:2), palmitic acid (C16:0) and caffeic acid were significantly negatively correlated with oxidative stability, and the content of linoleic acid (C18:2) increased with the ripening process.

The PERMANOVA results showed that varieties played a decisive role in the variation of oxidative stability, at 93.46%. *Koroneiki* was the cultivar with the highest oxidative stability at 40.16 h due to the highest level of oleuropein aglycone, oleacein, and TPP, while *Arbequina* held the lowest oxidative stability at 7.94 h with higher content of linoleic acid (C18:2) and lowest content of TPP, oleacein, oleic acid (C18:1) and oleuropein aglycone. The oxidative stability of *Zhongshan24* was 154.12% higher than that of the parent *Ascolana tenera*, due to the higher content of phenolics. Moreover, *Picual* recorded lower content of phenolics, but had a higher level of oxidative stability, due to having the highest percentage of oleic acid. In summary, the combined effect of FAs and phenolic components determined the oxidative stability of VOO.

### 3.3. Multivariate Analyses

**Figure 3:**
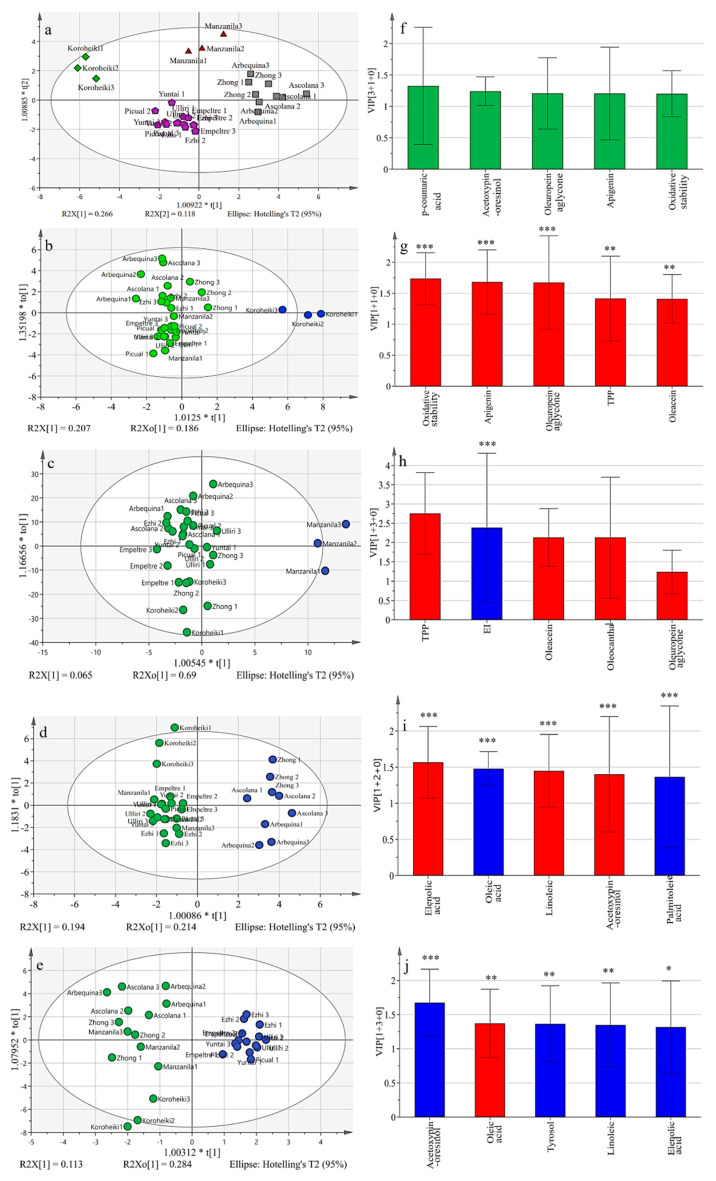
OPLS-DA models: ten varieties (**a**), ‘*Koroneiki*’ vs others (**b**), ‘*Manzanilla*’ vs others (**c**), ‘*Ascolana tenera*, *Arbequina* and *Zhongshan24*’ vs others (**d**), ’*Ezhi8, Ulliri Bardhe, Empeltre, Yuntai14* and *Picual*’ vs others (**e**); summary of the first five variables of the important differential variables in each left separation group, respectively (**f**–**j**). Red indicates a higher level and blue indicates a lower level. Green does not involve in level expression. “***”, *p* < 0.001, “**”, *p* < 0.01, “*”, *p* < 0.05. ^1^ Extractability index (EI); total phenolic content (TPP).

In order to further explore the relationship between olive varieties, the OPLS–DA model was used to differentiate the ten varieties. The model presented good reliability (R^2^Y = 0.917) and predictability (Q^2^ = 0.827) according to the 30 parameters mentioned above (5 physical indicators, 10 FAs and 13 phenolics, TPP, oxidative stability). Docosanoic acid and luteolin were removed to ensure model accuracy. Phenolic substances were the main factor in the differentiation of the ten varieties into four groups, and ‘each group vs others’ is presented in Figure 3. Meanwhile, the ‘each group vs others’ model indicated that R^2^Y and Q^2^ were consistently above 0.886 and 0.72, respectively (Table S6). The fitting degree of the model was verified by permutation analysis (Figure S2). VIP was used to obtain the most important variables of the ‘each group vs others’ model and the first five variables are summarized in Figure 3. Phenolics were the principal factor dividing the ten varieties into four groups. High phenolic levels were observed in the ‘*Koroneiki*’ group and the *‘Manzanilla’* group, but the oil extractability index differentiated them, being the highest and lowest, respectively. The ‘*Koroneiki*’ group demonstrated high oil productivity and oil quality, which was worthy of promotion in large-scale cultivation. The low oil extraction yield was a limiting factor for the *‘Manzanilla’* group, which was suggested to be improved through crossbreeding programs. The *‘Ascolana tenera, Arbequina and Zhongshan24’* group had high percentages of linoleic acid combined with gradually decreased oil extraction yield, and early harvesting was recommended for these cultivars to reduce the loss of oil oxidative stability. The *‘Ulliri Bardhe, Empeltre, Ezhi8, Yuntai14* and *Picual*’ group clustered for the higher relative value of oleic acid, together with gradually increased extraction yield during maturation.

**Figure 4:**
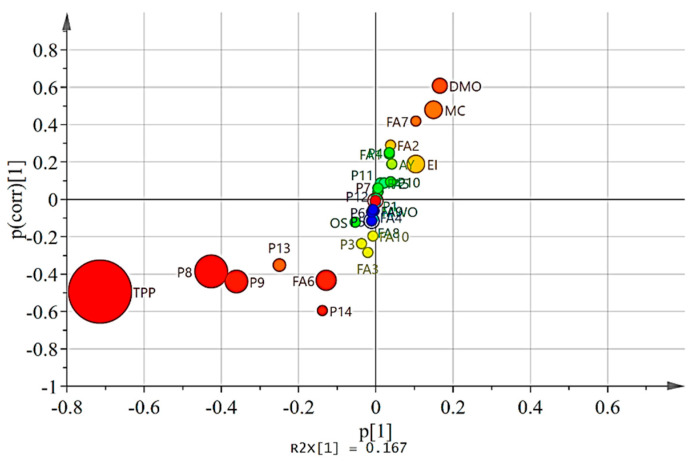
S-Plot providing the visualization of variables affected by maturity index. The circular size was positively correlated with the degree of influence. ^1^ Dry matter oil content (DMO); moisture content (MC); wet matter oil content (WMO); extractability index (EI); abencor yield (AY); palmitic acid (FA1); palmitoleic acid (FA2); heptadecanoic acid (FA3); heptadecenoic acid (FA4); stearic acid (FA5); oleic acid (FA6); linoleic (FA7); linolenic acid (FA8); arachidic acid (FA9); arachidonic acid (FA10); hydroxytyrosol (P1); tyrosol (P2); vanillic acid (P3); caffeic acid (P4); vanillin (P5); p-coumaric acid (P6); elenolic acid (P7); oleacein (P8); oleocanthal (P9); acetoxypinoresinol (P10); luteolin (P11); apigenin (P12); oleuropein aglycone(P13); ligstroside aglycone (P14); total phenolic content (TPP); oxidative stability (OS).

The OPLS model was constructed to investigate the effect of MI on these variables. The OPLS regression model was established by setting MI as variable Y and the 30 parameters as variable X. The model had good reliability (R^2^Y = 0.89) and predictability (Q^2^ = 0.778). The permutation analysis on model accuracy is presented in Figure S3. S-plot visualized the impact of MI on variables (Figure 4). It was observed that MI predominantly negative influenced the level of TPP, secoiridoids (oleacein, oleocanthal, oleuropein aglycone and ligstroside aglycone) and oleic acid. Meanwhile, MI positively affected the amount of WMO, MC, and EI. As for oxidative stability, MI had a limited negative impact.

## 4. Conclusions

To conclude, great variability in oil content, extraction yield, fatty acids, phenolics, and oxidative stability associated with VOO from ten cultivars grown in Wudu was found. MI most affected the level of oil content, oleic acid and secoiridoids. In addition, after genetic breeding improvement, compared with the parent variety *Ascolana tenera*, *Zhongshan24* VOOs significantly improved in oil quantity and quality. The chemical composition of VOOs in Wudu was in accordance with those in VOOs from Mediterranean countries, while the former had a higher level of PUFAs and a relatively lower content of oleuropein aglycone and ligstroside aglycone. The factors that cause differences in VOO characteristics and secondary metabolism need to be further discussed. The OPLS–DA model identified and classified varietal characteristics based on varietal parameters, which had positive implications for sensory regulation and harvesting in the VOO management process, as well as offering the possibility of varietal traceability. These findings contribute to varietal selection, improvement and harvesting in olive practice for local governments.

## Figures and Tables

**Table 1:** The information on olive varieties planted in Wudu.

Variety	Origin	Type	Variety	Origin	Type
*Yuntai14*	China	mid-ripening	*Ulliri Bardhe*	Albania	precocious-ripening
*Ezhi8*	China	slow-ripening	*Empeltre*	Spain	mid-ripening
*Zhongshan24*	China	precocious-ripening	*Ascolana tenera*	Italy	precocious-ripening
*Picual*	Spain	mid-ripening	*Manzanilla*	Spain	mid-ripening
*Arbequina*	Spain	mid-ripening	*Koroneiki*	Greece	slow-ripening

## Data Availability

The data presented in this study are available on request from the corresponding authors.

## Supplementary Materials

The following supporting information can be downloaded at: https://www.mdpi.com/article/10.3390/foods12010007/s1. Figure S1. Mass spectrogram of *Zhongshan24* virgin olive oils by LTQ-Orbitrap XL; Figure S2. Physical indicators of VOO from ten olive cultivars in Wudu (%); Figure S3. Fatty Acid composition of virgin olive oils from ten cultivars with three maturity indexes in Wudu (% of total composition) (a, b); Figure S4. Phenolic composition of virgin olive oils from ten cultivars with three maturity indexes in Wudu. (a) phenolic alcohols; (b) secoiridoids; (c) flavonoids; (d) phenolic acids and aldehydes; Figure S5. Oxidative stability of virgin olive oils from ten cultivars with three maturity indexes in Wudu; Table S1 Main phenolic compounds identified in a representative extract of *Zhongshan24* virgin olive oils by LTQ-Orbitrap XL; Table S2 The relative importance of varieties and maturity index and significance in the PERMANOVA for all the variables analyzed (expressed as percentages of total sum of squares, %); Table S3 Physical indicators of virgin olive oils from ten cultivars with three maturity indexes in Wudu (%); Table S4 Fatty Acid composition of virgin olive oils from ten cultivars with three maturity indexes in Wudu (% of total composition). Table S5 Phenolic composition and oxidative stability of virgin olive oils from ten cultivars with three maturity indexes in Wudu (phenolics content expressed as mg/kg; oxidative stability expressed as h); Table S6 Autofit results for Orthogonal Projections to Latent Structures Discriminant Analysis (OPLS-DA) models and OPLS models, according to VOO physicochemical indicators.
