# Supplementary material for: Effect of Variety and Maturity Index on the Physicochemical Parameters Related to Virgin Olive Oil from Wudu (China)
Source: Foods. 2022 Dec 20;12(1):7. doi: 10.3390/foods12010007 (PMC9818180; doi:10.3390/foods12010007)
Supplement: Supplementary file 1 [file foods-12-00007-s001.zip › foods-2034696-supplementary.pdf]

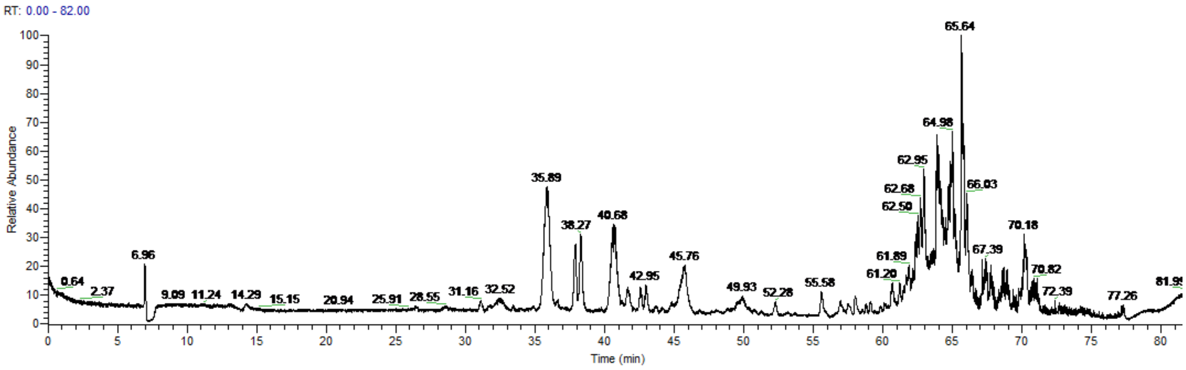

Figure S1. Mass spectrogram of *Zhongshan24* virgin olive oils by LTQ-Orbitrap XL.

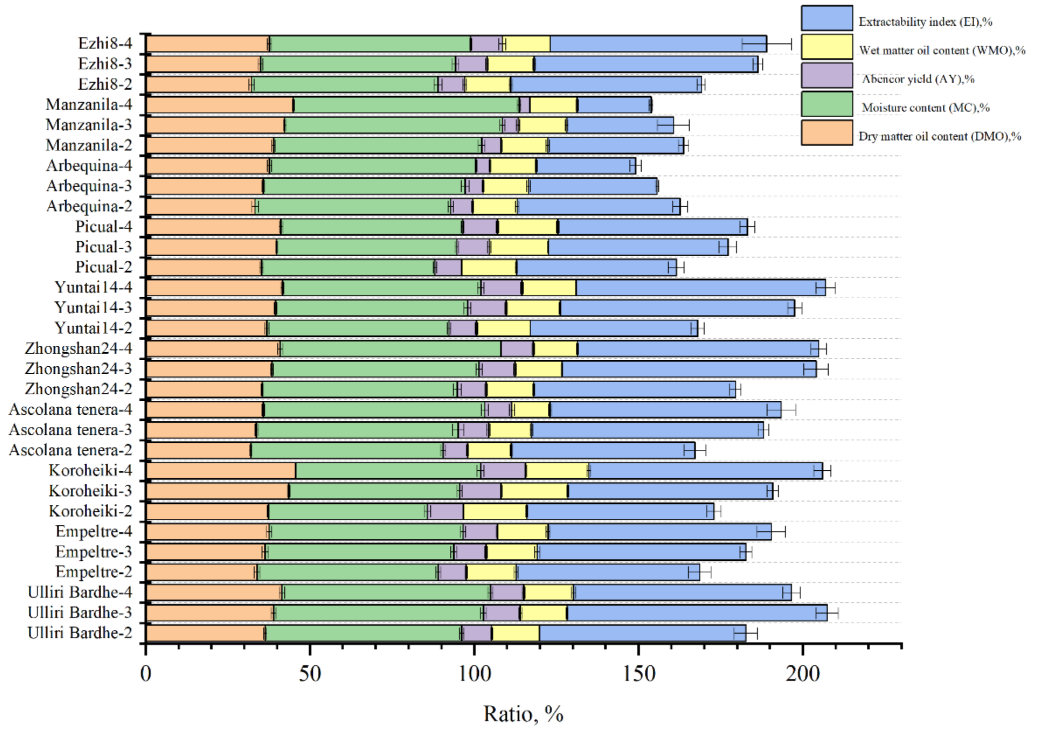

Figure S2. Physical indicators of VOO from ten olive cultivars in Wudu (%).

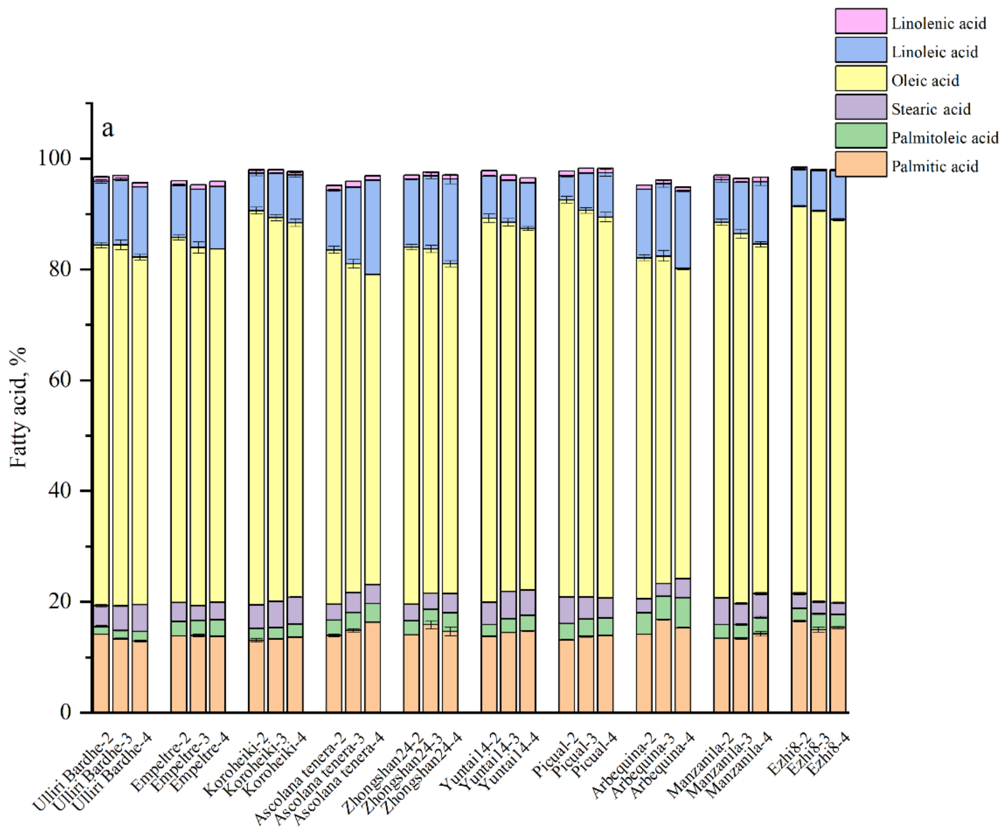

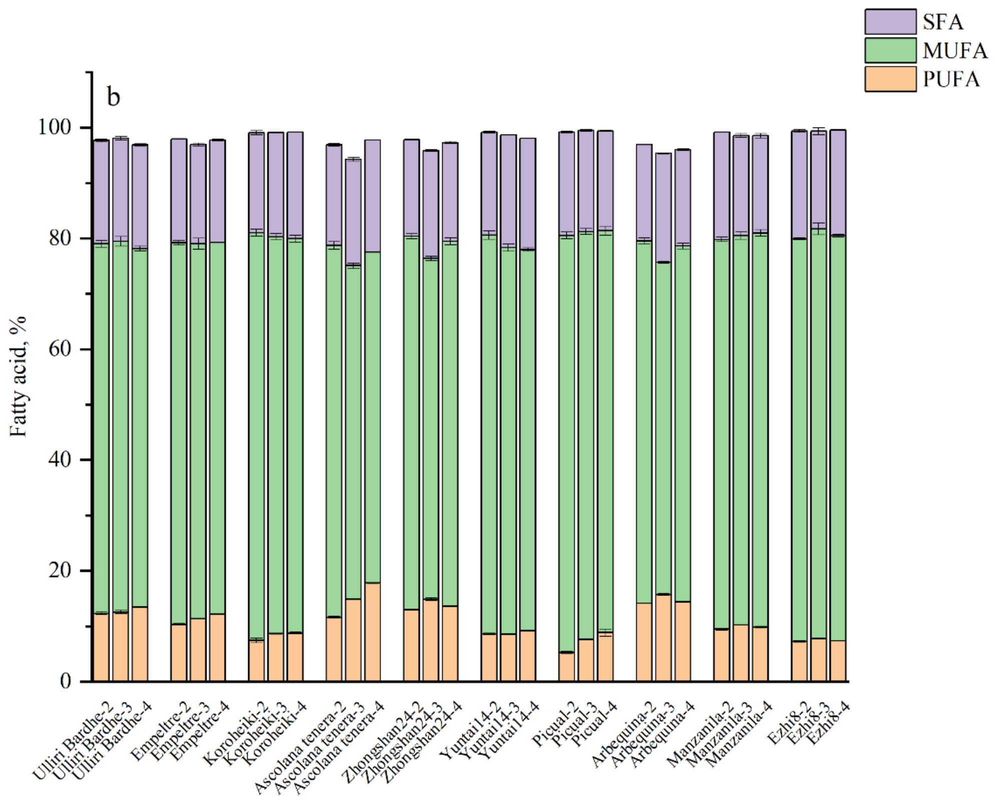

Figure S3. Fatty Acid composition of virgin olive oils from ten cultivars with three maturity indexes in Wudu (% of total composition) (a, b).

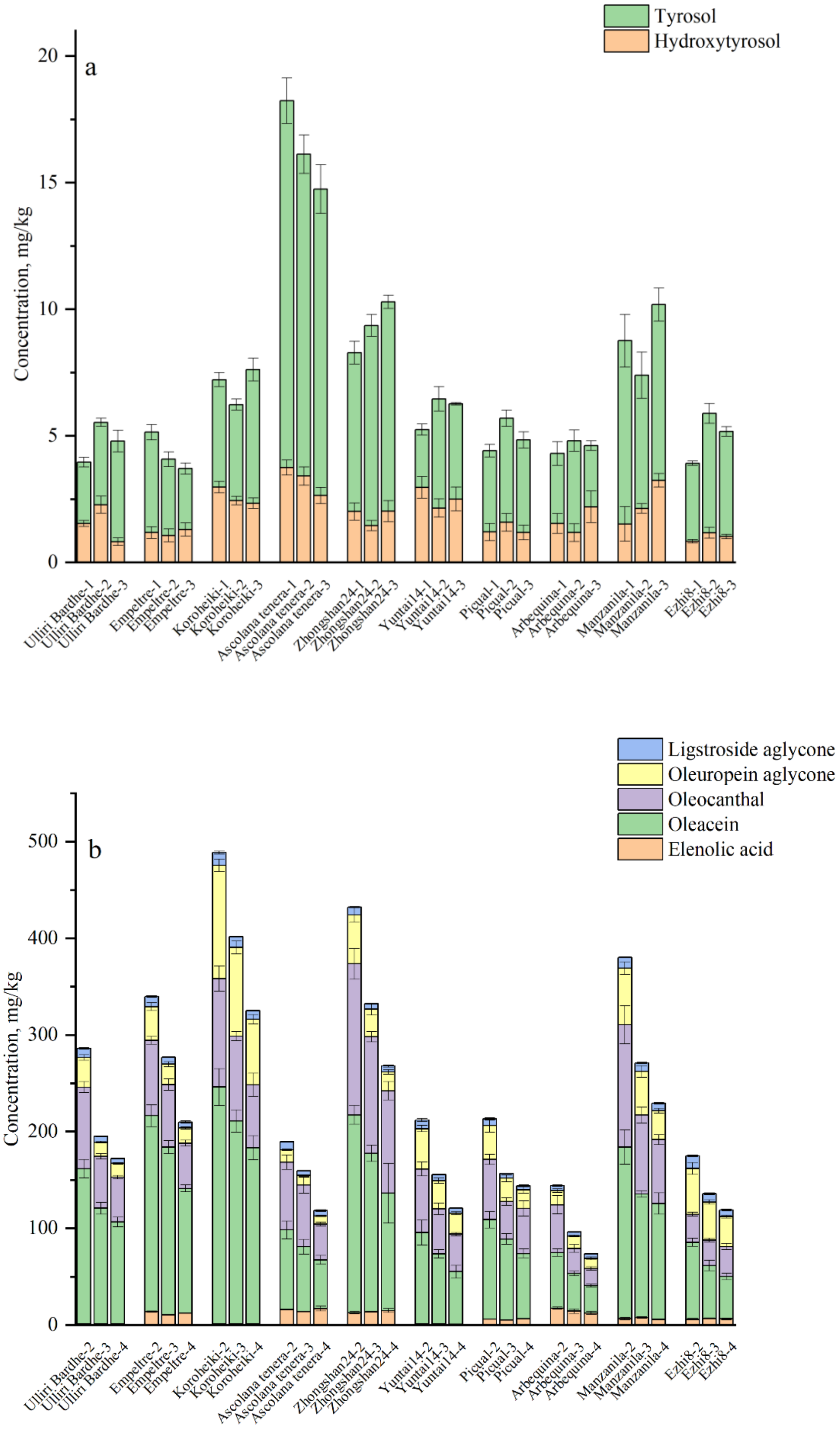
Tyrosol
Hydroxytyrosol
a
Concentration, mg/kg
0
5
10
15
20
Ulliri Bardhe-1
Ulliri Bardhe-2
Ulliri Bardhe-3
Empeltre-1
Empeltre-2
Empeltre-3
Koroheiki-1
Koroheiki-2
Koroheiki-3
Ascolana tenera-1
Ascolana tenera-2
Ascolana tenera-3
Zhongshan24-1
Zhongshan24-2
Zhongshan24-3
Yuntai14-1
Yuntai14-2
Yuntai14-3
Picual-1
Picual-2
Picual-3
Arbequina-1
Arbequina-2
Arbequina-3
Manzanila-1
Manzanila-2
Manzanila-3
Ezhi8-1
Ezhi8-2
Ezhi8-3
Ligstroside aglycone
Oleuropein aglycone
Oleocanthal
Oleacein
Elenolic acid
b
Concentration, mg/kg
0
100
200
300
400
500
Ulliri Bardhe-2
Ulliri Bardhe-3
Ulliri Bardhe-4
Empeltre-2
Empeltre-3
Empeltre-4
Koroheiki-2
Koroheiki-3
Koroheiki-4
Ascolana tenera-2
Ascolana tenera-3
Ascolana tenera-4
Zhongshan24-2
Zhongshan24-3
Zhongshan24-4
Yuntai14-2
Yuntai14-3
Yuntai14-4
Picual-2
Picual-3
Picual-4
Arbequina-2
Arbequina-3
Arbequina-4
Manzanila-2
Manzanila-3
Manzanila-4
Ezhi8-2
Ezhi8-3
Ezhi8-4

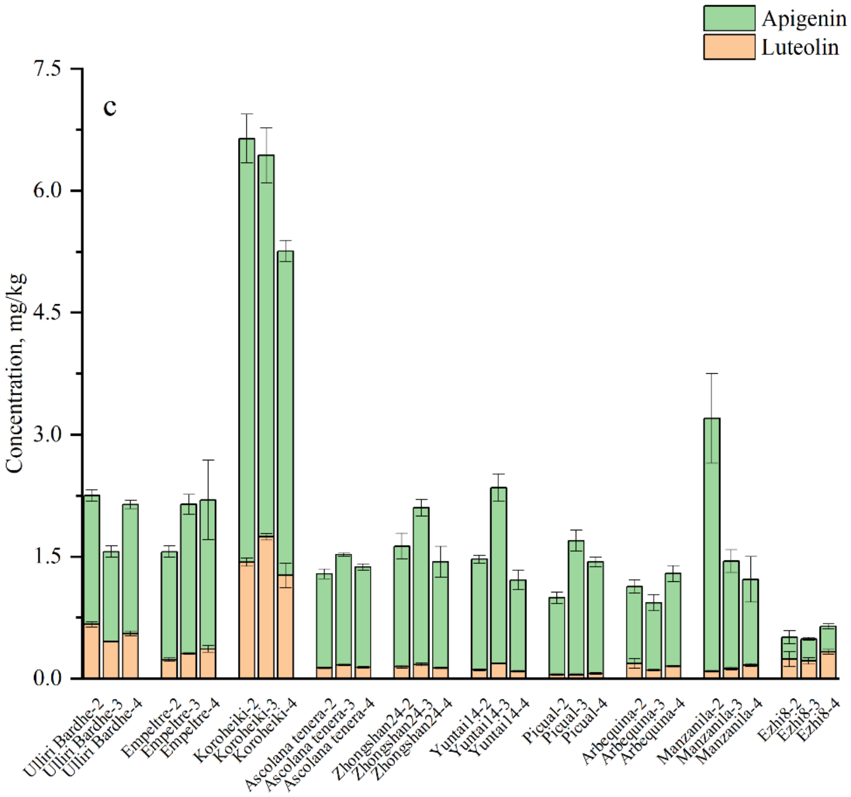
Apigenin
Luteolin
c
Concentration, mg/kg
7.5
6.0
4.5
3.0
1.5
0.0
Ulliri Bardhe-2
Ulliri Bardhe-3
Ulliri Bardhe-4
Empeltre-2
Empeltre-3
Empeltre-4
Koroheiki-2
Koroheiki-3
Koroheiki-4
Ascolana tenera-2
Ascolana tenera-3
Ascolana tenera-4
Zhongshan24-2
Zhongshan24-3
Zhongshan24-4
Yuntai14-2
Yuntai14-3
Yuntai14-4
Picual-2
Picual-3
Picual-4
Arbequina-2
Arbequina-3
Arbequina-4
Manzanila-2
Manzanila-3
Manzanila-4
Ezhi8-2
Ezhi8-3
Ezhi8-4

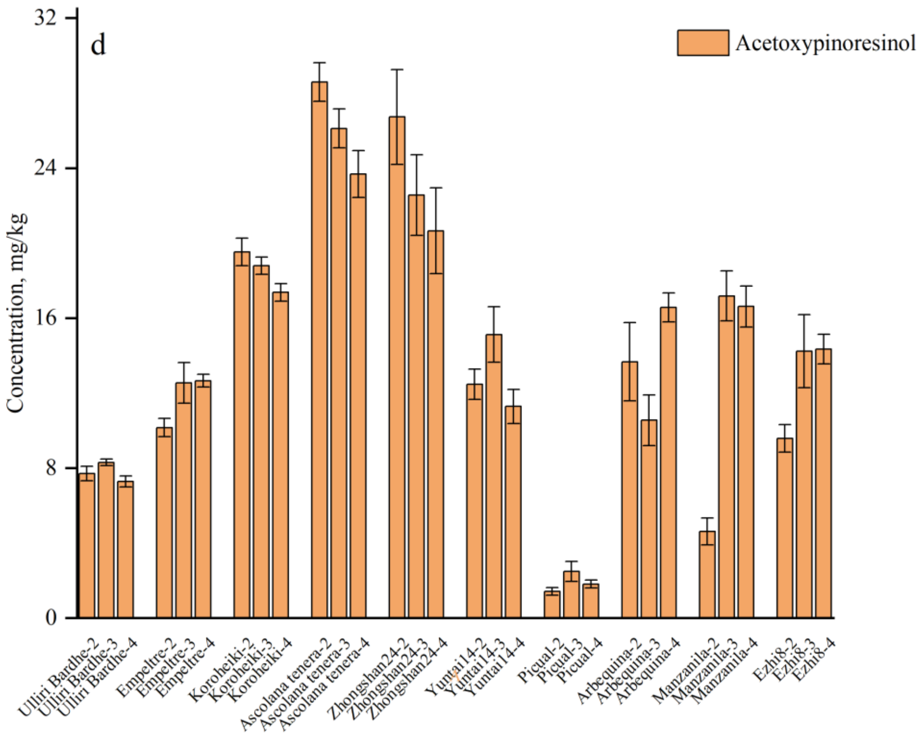
d
Acetoxypinoresinol
Concentration, mg/kg
32
24
16
8
0
Ulliri Bardhe-2
Ulliri Bardhe-3
Ulliri Bardhe-4
Empeltre-2
Empeltre-3
Empeltre-4
Koroheiki-2
Koroheiki-3
Koroheiki-4
Ascolana tenera-2
Ascolana tenera-3
Ascolana tenera-4
Zhongshan24-2
Zhongshan24-3
Zhongshan24-4
Yuntai14-2
Yuntai14-3
Yuntai14-4
Picual-2
Picual-3
Picual-4
Arbequina-2
Arbequina-3
Arbequina-4
Manzanila-2
Manzanila-3
Manzanila-4
Ezhi8-2
Ezhi8-3
Ezhi8-4

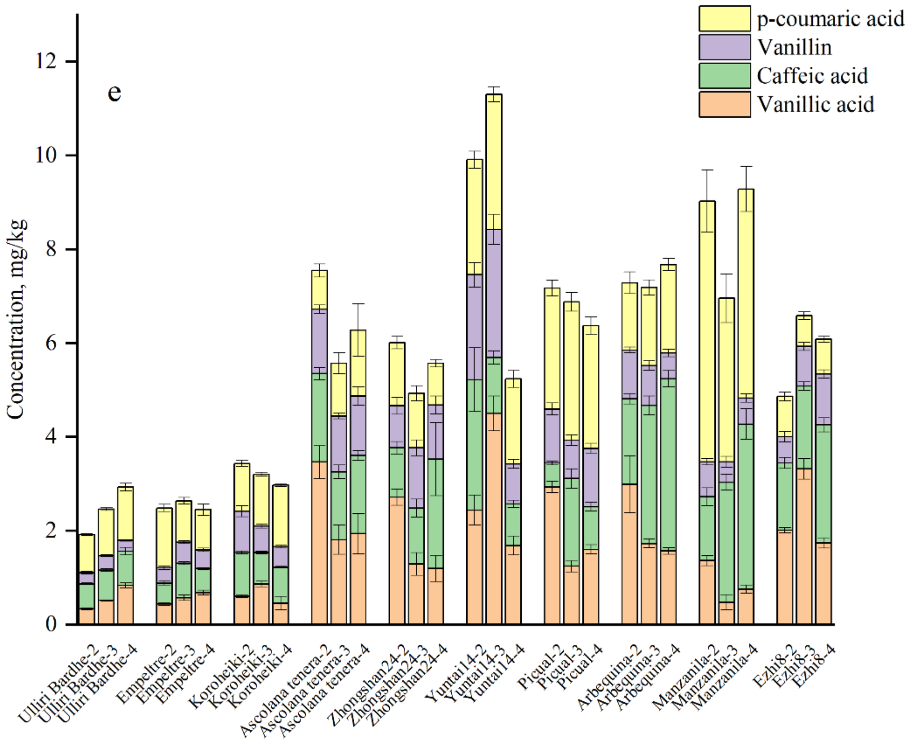

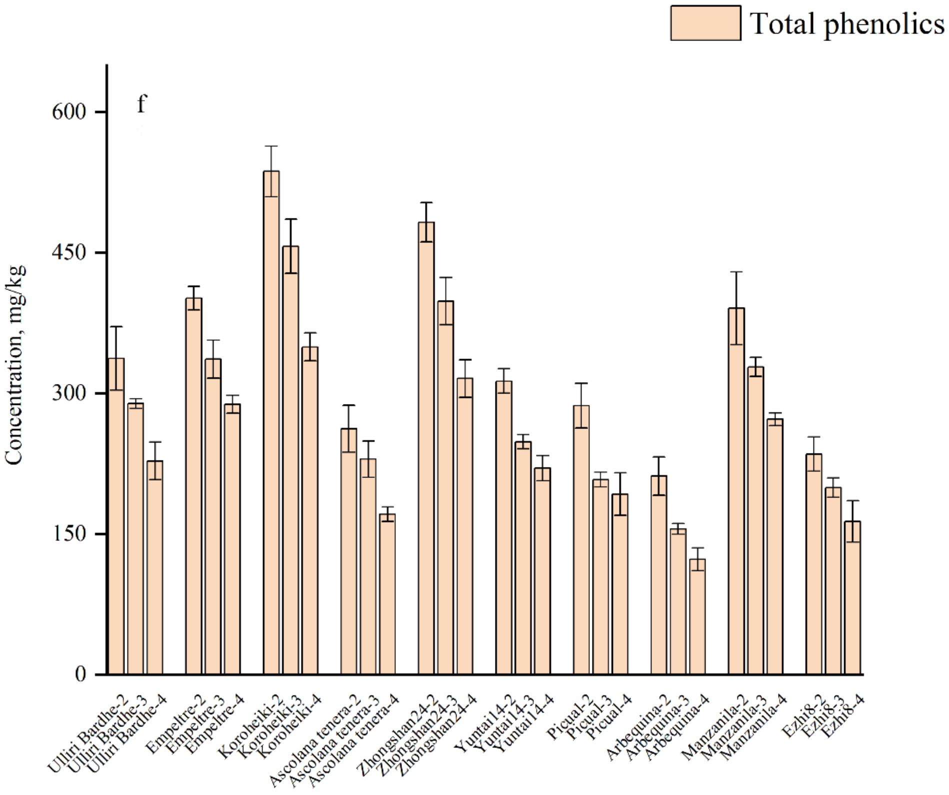

Figure S4. Phenolic composition of virgin olive oils from ten cultivars with three maturity indexes in Wudu. (a) phenolic alcohols; (b) secoiridoids; (c) flavonoids; (d) phenolic acids and aldehydes;

(e)lignans; (f) total phenolics.

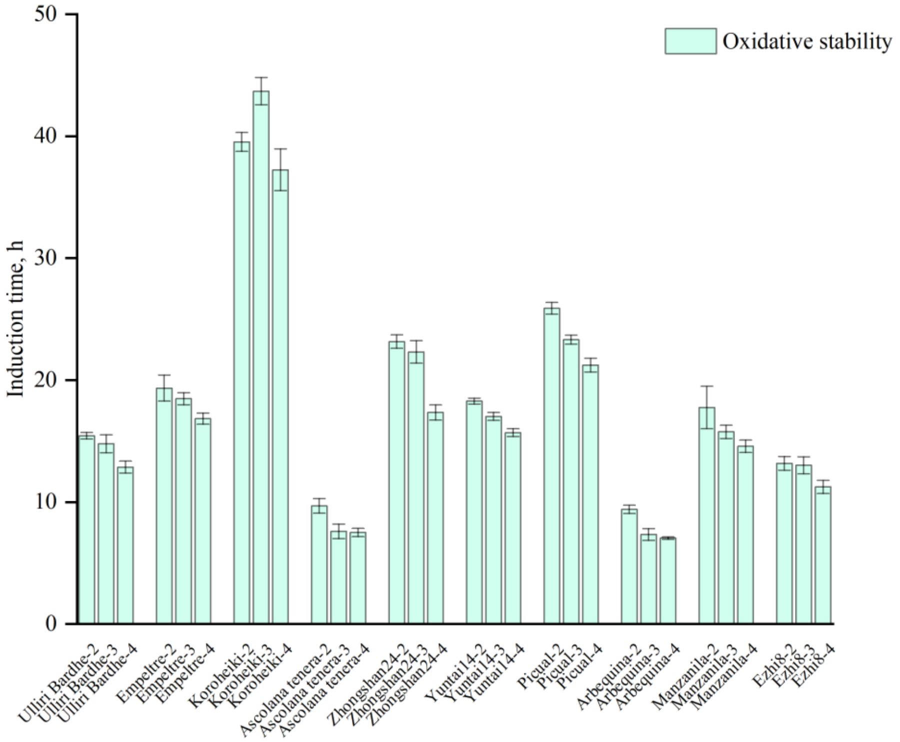

Figure S5. Oxidative stability of virgin olive oils from ten cultivars with three maturity indexes in Wudu.

Table S1. Main phenolic compounds identified in a representative extract of *Zhongshan24* virgin olive oils by LTQ-Orbitrap XL.

Peak number	Compounds	Retention time	Molecular formula	m/z calcd[a]	Calibration curve
1	Hydroxytyrosol	14.29	$C_8H_{10}O_3$	153.06299	y = 3141.4x - 1661.1
2	Tyrosol	17.56	$C_8H_{10}O_2$	137.06808	y = 2329.4x - 451
3	Caffeic acid	21.84	$C_9H_8O_4$	179.04226	y = 8843.9x - 847.95
4	Vanillic acid	22.25	$C_8H_8O_4$	167.04226	y = 1520.5x - 536.72
5	Vanillin	23.99	$C_8H_8O_3$	151.04734	y= 8846.7x – 1273.8
6	p-coumaric acid	26.77	$C_9H_8O_3$	163.04734	y = 4142x - 1168.2
7	Elenolic acid	32.52	$C_{11}H_{14}O_6$	241.07904	y = 7470x - 1498.9
8	Oleacein	35.89,38.27	$C_{17}H_{20}O_6$	319.12599	y = 33747.1x - 7881.09

9	Oleocanthal	40.68	$C_{17}H_{20}O_5$	303.13107	y = 314486x - 9458.9
10	Acetoxypinoresinol (In terms of pinoresinol)	41.63	$C_{22}H_{24}O_8$	415.14712	y = 14554x + 7209.8
11	Luteolin	44.32	$C_{15}H_{10}O_6$	285.04774	y = 1884x - 482.4
12	Oleuropein aglycone	41.58,45.76	$C_{19}H_{22}O_8$	377.13147	y = 25712x + 74649
13	Apigenin	48.96	$C_{15}H_{10}O_5$	269.05282	y = 3271x + 2497.1
14	Ligstroside aglycone	49.93	$C_{19}H_{22}O_7$	361.12900	y = 3764.5x – 696.91

[a]m/z calcd: calculated mass

Table S2. The relative importance of varieties and maturity index and significance in the PERMANOVA for all the variables analyzed (expressed as percentages of total sum of squares, %).

Variables	Cultivar	MI	Interaction	Error
DMO[1]	57.5***	36.97***	3.09	2.44
MC	74.22***	18.58***	3.74	3.46
AY	71.11***	13.34**	12.28	3.28
WMO	88.79***	0.05	4.71	6.44
EI	63.49***	15.79***	16.29	4.43
Palmitic acid	66.64***	4.61 n.s.	23.87 n.s.	4.88
Palmitoleic acid	80.76***	8.25*	9.62 n.s.	1.38
Heptadecanoic acid	77.22***	4.17 n.s.	17.78 n.s.	0.71
Heptadecenoic acid	82.63***	1.29 n.s.	8.76 n.s.	7.32
Stearic acid	81.72***	1.14 n.s.	16.21 n.s.	0.93
Oleic acid	83.02***	12.38**	2.59 n.s.	2.01
Linoleic	83.02***	12.29**	3.11 n.s.	1.58
Linolenic acid	87.38***	0.89 n.s.	6.54 n.s.	5.17
Arachidic acid	75.89***	0.25 n.s.	22.84 n.s.	0.99
Arachidonic acid	64.17***	2.12 n.s.	31.05 n.s.	2.63
Docosanoic acid	79.84***	0.22 n.s.	16.5 n.s.	3.27
PUFA	90.4***	4.56 n.s.	4.22 n.s.	0.81
MUFA	88.71***	4.13 n.s.	5.23 n.s.	1.92
SFA	23.48**	2.54 n.s.	68.89***	5.08
MUFA/PUFA	89.62***	5.53 n.s.	3.9 n.s.	0.95
Hydroxytyrosol	66.32***	0.19 n.s.	22.21 n.s.	11.29
Tyrosol	75.4***	2.88 n.s.	17.93 n.s.	3.79
Vanillic acid	75.02***	3.77 n.s.	18.53 n.s.	2.68
Caffeic acid	71.07***	2.71 n.s.	24.28 n.s.	1.94
Vanillin	77.64***	0.87 n.s.	17.35 n.s.	4.14
p-coumaric acid	82.58***	0.01 n.s.	11.42 n.s.	5.98
Elenolic acid	91.48***	0.03 n.s.	5.51 n.s.	2.98
Oleacein	84.85***	12.09**	0.75 n.s.	2.32
Oleocanthal	69.08***	19.58***	7.32 n.s.	4.03
Acetoxypinoresinol	84.1***	1.69 n.s.	12.39 n.s.	1.82
Luteolin	85.16***	0.74 n.s.	10.88 n.s.	3.18
Apigenin	65.3***	1.65 n.s.	27.6 n.s.	5.45
Oleuropein aglycone	77.59***	15.68***	4.04 n.s.	2.69
Ligstroside aglycone	56.1***	29.8***	6.96 n.s.	7.15
TPP	72.57***	17.72***	6.32 n.s.	3.39
Oxidative stability	93.46***	4.16 n.s.	1.32 n.s.	1.06

[1]maturity index (MI), dry matter oil content (DMO), moisture content (MC), wet matter oil content (WMO), extractability index (EI), abencor yield (AY), saturated fatty acid (SFA), polyunsaturated FA (PUFA), monounsaturated fatty acid (MUFA), total polar phenolics (TPP), permutational multivariate analysis of variance (PERMANOVA).

[2] *** = p-value < 0.001; ** = p-value < 0.01; * = p-value < 0.05; n.s. = not significant difference.

Table S3. Physical indicators of virgin olive oils from ten cultivars with three maturity indexes in Wudu (%).

Cultivar	MI[1]	DMO	MC	AY	MWO	EI
Ulliri Bardhe	2	36.45±0.47c	59.81±0.66b	9.05±0.3c	14.65±0.05ab	62.73±3.53b
Ulliri Bardhe	3	38.95±0.67a	63.9±0.83a	11.23±0.45a	14.19±0.31b	79.17±3.36a
Ulliri Bardhe	4	41.55±0.84b	63.46±0.8a	10.07±0.44b	15.19±0.54a	66.32±2.64b
Empeltre	2	33.92±0.86b	55.18±0.7c	8.48±0.28b	15.21±0.58	55.86±3.41b
Empeltre	3	36.4±0.98a	57.4±0.89b	9.85±0.35a	15.51±0.74	63.56±1.84a
Empeltre	4	37.62±0.78a	59±0.75a	10.47±0.3a	15.43±0.6	67.94±4.38a
Koroheiki	2	37.37±0.26c	48.42±0.9b	10.98±0.22c	19.28±0.35	56.98±2.1c
Koroheiki	3	43.57±0.34b	52.01±0.71a	12.64±0.3b	20.27±0.23	62.39±1.74b
Koroheiki	4	45.68±0.16a	56.31±1.02a	13.67±0.3a	19.23±0.64	71.15±2.63a
Ascolana tenera	2	32.04±0.36c	58.58±0.79c	7.41±0.31b	13.27±0.34a	55.87±3.25b
Ascolana tenera	3	33.77±0.47b	61.4±1.76b	9.21±0.5a	13.03±0.43ab	70.66±1.7a
Ascolana tenera	4	35.79±0.42a	67.49±1.03a	8.2±0.82sb	11.63±0.46b	70.34±4.34a
Zhongshan24	2	35.49±0.3c	59.35±1.17c	8.86±0.32c	14.43±0.33	61.41±1.72c
Zhongshan24	3	38.49±0.42b	62.99±0.88b	11.01±0.39a	14.24±0.19	77.34±3.72a
Zhongshan24	4	40.98±0.71a	67.17±0.18a	9.88±0.29b	13.45±0.3	73.43±2.39b
Yuntai14	2	36.88±0.72c	55.53±0.6c	8.34±0.39c	16.4±0.1	50.87±2.07b
Yuntai14	3	39.57±0.36b	58.41±1.01b	11.75±0.3b	16.46±0.25	71.39±2.13a
Yuntai14	4	41.67±0.46a	60.38±0.96a	12.53±0.38a	16.51±0.22	75.9±2.99a
Picual	2	35.3±0.54c	52.79±0.5b	8.11±0.2b	16.67±0.41b	48.68±2.35b
Picual	3	39.92±0.2b	54.83±0.39b	9.85±0.56a	18.03±0.15a	54.61±2.69a
Picual	4	41.15±0.53a	55.38±0.37a	10.59±0.36a	18.36±0.37a	57.71±2.3a
Arbequina	2	33.35±1.01c	59.53±0.83b	6.7±0.33a	13.5±0.51	49.66±2.25a
Arbequina	3	35.84±0.32b	61.46±1.16a	5.42±0.27b	13.81±0.53	39.2±0.5b
Arbequina	4	37.73±0.59a	62.85±0.31a	4.25±0.17c	14.02±0.32	30.32±1.74c
Manzanila	2	38.95±0.51c	63.37±1.02c	5.89±0.25a	14.27±0.53	41.29±1.56a
Manzanila	3	42.39±0.36b	66.27±0.82b	4.78±0.57b	14.75±0.48	32.49±4.84b
Manzanila	4	44.96±0.33a	68.83±0.52a	3.23±0.09c	14.33±0.31	22.55±0.58c
Ezhi8	2	32.32±0.84c	56.7±1.23c	8.13±0.46b	13.99±0.54	58.05±1.23b
Ezhi8	3	35.01±0.74b	59.32±1.07b	9.7±0.42a	14.25±0.49	68.11±1.46a
Ezhi8	4	37.68±0.57a	61.3±0.3a	9.61±1.05ab	14.58±0.14	65.91±7.5ab
Cultivar		DMO	MC	AY	MWO	EI
Ulliri Bardhe	Mean values	38.98±2.28abcd	62.39±2.05abc	10.12±1.01ab	14.67±0.53de	69.41±7.98ab
Empeltre	Mean values	35.98±1.8bcd	57.19±1.8cde	9.6±0.92b	15.38±0.57d	62.45±6.05ab
Koroheiki	Mean values	42.21±3.75a	52.25±3.51e	12.43±1.2a	19.59±0.64a	63.51±6.47ab
Ascolana tenera	Mean values	33.87±1.66d	62.49±4.1abc	8.27±0.93b	12.65±0.84f	65.62±7.85ab
Zhongshan24	Mean values	38.32±2.42abcd	63.17±3.47ab	9.92±0.98ab	14.04±0.51e	70.73±7.57a
Yuntai14	Mean values	39.37±2.13abc	58.11±2.25bcd	10.87±1.95ab	16.45±0.18c	66.06±11.74ab
Picual	Mean values	38.79±2.7abcd	54.33±1.24de	9.52±1.16b	17.69±0.83b	53.67±4.51bc
Arbequina	Mean values	35.64±2cd	61.28±1.62abc	5.45±1.09c	13.78±0.46e	39.72±8.51cd
Manzanila	Mean values	42.10±2.63ab	66.15±2.47a	4.63±1.2c	14.45±0.45de	32.11±8.52d
Ezhi8	Mean values	35±2.4cd	59.1±2.16bcd	9.15±0.98b	14.27±0.45e	64.02±6ab

[1] Maturity index (MI), dry matter oil content (DMO), moisture content (MC), wet matter oil content (WMO), extractability index (EI), abencor yield (AY).

[2]Letters "a, b, c" indicates significant differences at a level of $p < 0.05$ by Tukey's test between MI, within the same cultivar.

[3]Different letters indicated the significant differences at a level of $p < 0.05$ by Tukey's test within the ten cultivar.

Table S4. Fatty Acid composition of virgin olive oils from ten cultivars with three maturity indexes in Wudu (% of total composition).

Cultivar	MI	Palmitic acid	Palmitoleic acid	Heptadecanoic acid	Heptadecenoic acid	Stearic acid	Oleic acid	Linoleic	Linolenic acid	Arachidic acid	Arachidonic acid	Docosanoic acid	PUFA[1]	MUFA	SFA	MUFA/PUFA
Ulliri Bardhe	2	14.15±0.06[2]a	1.39±0.15b	0.28±0.02c	ND	3.75±0.28c	65.1±0.52a	11.51±0.34b	0.79±0.12	0.39±0.03c	0.22±0.03	0.11±0.04	12.3±0.23b	66.7±0.59a	18.67±0.28	5.42±0.13a
Ulliri Bardhe	3	13.29±0.16b	1.51±0.07b	0.32±0.01b	ND	4.45±0.14b	65.17±0.86a	11.8±0.24b	0.74±0.03	0.47±0.01b	0.24±0.01	0.09±0.01	12.54±0.27b	66.93±0.89a	18.62±0.31	5.34±0.19a
Ulliri Bardhe	4	12.89±0.18c	1.76±0.04a	0.34±0.01a	ND	4.86±0.02a	62.71±0.49c	12.7±0.07a	0.75±0.01	0.53±0.01a	0.25±0.01	0.11±0.01	13.44±0.07a	64.73±0.45b	18.73±0.17	4.82±0.02b
Empeltre	2	13.83±0.09a	2.64±0.02c	0.94±0.02a	ND	3.36±0.02a	65.91±0.43a	9.49±0.15c	0.79±0.01b	0.46±0.01b	0.39±0.01a	0.12±0.01a	10.29±0.15c	68.94±0.42a	18.72±0.08a	6.7±0.13a
Empeltre	3	13.91±0.21a	2.74±0.01b	0.85±0.01b	ND	2.67±0.02c	64.64±0.98ab	10.53±0.05b	0.79±0.01b	0.39±0.01c	0.34±0.01c	0.09±0.02b	11.32±0.04b	67.71±0.97ab	17.91±0.24b	5.98±0.06b
Empeltre	4	13.78±0.12a	2.97±0.01a	0.96±0.01a	ND	3.16±0.02b	63.8±0.06b	11.25±0.06a	0.91±0.01a	0.45±0.01a	0.36±0.01b	0.11±0.01a	12.16±0.07a	67.14±0.07b	18.46±0.11a	5.52±0.04c
Koroheiki	2	13.03±0.29b	2.17±0.01b	0.23±0.03a	ND	4.23±0.08c	71.17±0.62a	6.76±0.45b	0.67±0.02a	0.47±0.01b	0.24±0.02b	0.1±0.01c	7.43±0.43b	73.58±0.61b	18.06±0.39b	9.93±0.63b
Koroheiki	3	13.25±0.08ab	2.08±0.03c	0.24±0.01a	ND	4.74±0.03b	69.26±0.59b	8.01±0.08a	0.64±0.01a	0.52±0.01a	0.28±0.01a	0.11±0.01b	8.65±0.09a	71.62±0.59b	18.86±0.07a	8.28±0.15b
Koroheiki	4	13.59±0.11a	2.36±0.01a	0.13±0.01b	ND	4.88±0.08a	67.59±0.64c	8.68±0.37a	0.61±0.01b	0.53±0.01a	0.28±0.01a	0.12±0.01a	8.75±0.14a	71.23±0.64a	19.24±0.03a	8.14±0.2a
Ascolana tenera	2	13.91±0.26c	2.81±0.03c	0.89±0.01a	0.31±0.58b	2.84±0.01c	63.98±0.66a	11.76±0.16c	0.84±0.01b	0.41±0.01b	0.35±0.01a	0.1±0.01b	12.6±0.17c	67.14±0.68a	18.14±0.25c	5.36±0.11a
Ascolana tenera	3	14.82±0.3b	3.22±0.04b	0.15±0.01b	0.33±0.01a	3.61±0.05a	59.38±0.78b	14.84±0.07b	1.05±0.01a	0.47±0.01a	0.27±0.01b	0.12±0.01a	15.89±0.07b	60.2±0.45b	19.16±0.35b	3.98±0.04b
Ascolana tenera	4	16.32±0.08a	3.36±0.02a	0.1±0.01c	0.24±0.01c	3.42±0.02b	56.00±0.05c	18.34±0.55a	0.79±0.01c	0.29±0.01c	0.16±0.01c	0.06±0.01c	19.13±0.1a	59.76±0.05b	20.19±0.07a	3.13±0.09c
Zhongshan24	2	14.01±0.07b	2.58±0.03c	0.09±0.01a	0.22±0.01a	2.99±0.02b	64.44±0.49a	12.28±0.12a	0.69±0.01a	0.29±0.01a	0.17±0.01a	0.06±0.01a	12.98±0.13c	67.41±0.46a	17.43±0.08c	5.2±0.08a
Zhongshan24	3	15.86±0.7a	2.77±0.01b	0.08±0.01b	0.2±0.01b	2.9±0.02c	62.14±0.63b	13.24±1.54c	0.64±0.01c	0.24±0.01c	0.15±0.01c	0.05±0.01c	14.84±0.22a	61.55±0.4c	19.47±0.16a	4.15±0.04c
Zhongshan24	4	14.64±0.78ab	3.38±0.03a	0.09±0.01a	0.22±0.01a	3.46±0.05a	59.53±0.57c	15.33±0.86b	0.67±0.01b	0.28±0.01b	0.16±0.01b	0.05±0.01b	13.58±0.07b	65.89±0.64b	17.82±0.24b	4.85±0.06b
Yuntai14	2	13.78±0.09c	2.08±0.02c	0.14±0.01a	0.26±0.01c	4.04±0.08c	69.32±0.75a	7.64±0.1a	0.95±0.02a	0.52±0.01c	0.33±0.01c	0.11±0.01b	8.59±0.11b	71.99±0.73b	18.58±0.17c	8.38±0.19a
Yuntai14	3	14.46±0.07b	2.45±0.02b	0.16±0.01b	0.3±0.01a	4.93±0.06a	66.65±0.67b	7.57±0.13b	0.96±0.01a	0.64±0.01a	0.4±0.01a	0.14±0.01a	8.54±0.13b	69.8±0.66b	20.34±0.07a	8.18±0.11a
Yuntai14	4	14.73±0.08a	2.85±0.06a	0.14±0.01a	0.28±0.01b	4.49±0.02b	65.32±0.37c	8.24±0.08b	0.91±0.02b	0.61±0.01b	0.37±0.01b	0.14±0.01a	9.15±0.08a	68.81±0.32a	20.12±0.05b	7.52±0.1b
Picual	2	12.1±0.11b	2.97±0.02b	0.18±0.01a	0.37±0.09	4.8±0.03a	71.67±0.59a	5.34±0.16c	0.89±0.02a	0.49±0.01a	0.31±0.01a	0.1±0.01a	6.23±0.17c	75.32±0.62a	17.67±0.14a	12.21±0.43a
Picual	3	13.76±0.15a	3.14±0.02a	0.15±0.01b	0.39±0.01	3.94±0.03b	69.81±0.51b	6.77±0.08b	0.86±0.01b	0.38±0.01b	0.26±0.01b	0.07±0.01b	7.63±0.09b	73.6±0.52b	18.3±0.18b	9.65±0.09b
Picual	4	13.87±0.07a	3.2±0.06a	0.13±0.01c	0.33±0.01	3.58±0.02c	68.79±0.84b	8.01±0.63a	0.82±0.01c	0.37±0.01c	0.24±0.01c	0.07±0.01b	8.83±0.64a	72.56±0.8b	18.02±0.08b	8.26±0.71c
Arbequina	2	14.12±0.03c	3.9±0.06c	0.25±0.01a	0.66±0.01a	2.5±0.02b	61.55±0.48a	12.4±0.07b	0.74±0.01b	0.41±0.01a	0.3±0.01a	0.1±0.01a	14.14±0.07c	65.4±0.54a	17.38±0.05b	4.98±0.05a
Arbequina	3	16.8±0.09a	4.15±0.05b	0.19±0.01c	0.56±0.01c	2.34±0.02c	59.13±0.96b	13.03±0.59b	0.67±0.01b	0.3±0.01c	0.23±0.01c	0.07±0.01c	15.72±0.13a	59.94±0.15c	19.71±0.08a	4.07±0.04c
Arbequina	4	15.27±0.1b	5.44±0.05a	0.22±0.01b	0.64±0.01b	3.43±0.02a	56.00±0.17c	14.05±0.13a	0.68±0.01a	0.38±0.01b	0.25±0.01b	0.09±0.01b	14.37±0.12b	64.26±0.52b	17.38±0.1b	4.81±0.08b
Manzanila	2	13.42±0.03b	2.47±0.04b	0.45±0.01a	0.93±0.02a	4.82±0.08a	67.8±0.62a	7.71±0.44c	0.77±0.01	0.68±0.03a	0.3±0.01a	0.39±0.02a	9.45±0.16c	70.34±0.41	19.38±0.05a	7.44±0.15a
Manzanila	3	13.38±0.2b	2.48±0.14b	0.35±0.03b	0.83±0.02b	3.84±0.12b	66.72±0.85a	9.3±0.1b	0.72±0.09	0.51±0.02b	0.23±0.01	0.27±0.01b	10.25±0.03a	70.25±0.68	18.08±0.35b	6.86±0.08b

Manzanila	4	14.2±0.42a	2.95±0.23a	0.29±0.02c	0.63±0.07c	4.31±0.21c	63.09±0.47b	11.31±0.71a	0.8±0.07	0.43±0.03c	0.22±0.02b	0.24±0.01c	9.83±0.17b	71.14±0.58	17.57±0.42b	7.24±0.11a
Ezhi8	2	16.49±0.13a	2.34±0.09b	0.2±0.01b	0.23±0.02c	2.64±0.22a	69.92±0.17	6.68±0.12c	0.4±0.01a	0.18±0.01c	0.16±0.02c	0.14±0.01c	7.24±0.14b	72.65±0.19a	19.51±0.27a	10.04±0.18a
Ezhi8	3	14.98±0.48b	2.83±0.1a	0.25±0.01a	0.33±0.02b	2.19±0.15b	70.56±1.14	7.23±0.11b	0.27±0.01b	0.24±0.05b	0.24±0.01b	0.22±0.01b	7.74±0.09a	73.96±1.03b	17.67±0.65b	9.56±0.24b
Ezhi8	4	15.39±0.25b	2.28±0.03b	0.2±0.01b	0.41±0.02a	2.12±0.15b	69.18±0.21	8.82±0.07a	0.26±0.01b	0.36±0.01a	0.28±0.01a	0.27±0.01a	7.36±0.07b	73.15±0.2a	19.06±0.11a	9.94±0.12ab
Cultivar		Palmitic acid	Palmitoleic acid	Heptadecanoic acid	Heptadecenoic acid	Stearic acid	Oleic acid	Linoleic	Linolenic acid	Arachidic acid	Arachidonic acid	Docosanoic acid	PUFA[1]	MUFA	SFA	MUFA/PUFA
Ulliri Bardhe		13.44±0.57[3]d	1.55±0.19f	0.31±0.03b	ND	4.35±0.51ab	64.33±1.33de	12±0.57bc	0.76±0.07cde	0.47±0.06bcd	0.24±0.02b	0.1±0.02bc	12.76±0.55bc	66.12±1.2cd	18.68±0.23b	5.19±0.31de
Empeltre		13.84±0.14d	2.78±0.15bcde	0.92±0.05a	ND	3.06±0.31d	64.78±1.06cde	10.42±0.77cd	0.83±0.06bcd	0.43±0.04bcd	0.36±0.02a	0.11±0.01bc	11.26±0.82cd	67.93±0.95bc	18.36±0.38b	6.07±0.52cd
Koroheiki		13.29±0.29d	2.2±0.12e	0.2±0.05bcd	ND	4.62±0.3ab	69.34±1.64abc	7.82±0.89de	0.64±0.03e	0.5±0.03ab	0.27±0.02b	0.11±0.01bc	8.28±0.68ef	72.14±1.21ab	18.72±0.56b	8.78±0.92ab
Ascolana tenera		15.01±1.08ab	3.13±0.25bc	0.38±0.39bcd	0.29±0.15cd	3.29±0.35cd	59.79±3.51f	14.98±2.7a	0.89±0.12ab	0.39±0.08de	0.26±0.08b	0.09±0.02bc	15.87±2.69a	63.50±3.61e	19.16±0.91ab	4.16±0.98e
Zhongshan24	Mean values	14.83±0.97abc	2.91±0.36bcd	0.09±0.01d	0.21±0.01cd	3.12±0.26cd	62.04±2.18ef	13.62±1.61ab	0.67±0.02e	0.27±0.02ef	0.16±0.01c	0.05±0.01c	13.8±0.84ab	64.95±2.67cde	18.24±0.95b	4.73±0.47de
Yuntai14		14.33±0.43bcd	2.46±0.33de	0.15±0.01cd	0.28±0.02cd	4.49±0.39a	67.1±1.84abcd	7.82±0.33e	0.94±0.03a	0.59±0.06a	0.37±0.03a	0.13±0.02b	8.76±0.31ef	70.2±1.5ef	19.68±0.83a	8.03±0.41ab
Picual		13.24±0.86d	3.1±0.11b	0.15±0.02cd	0.36±0.05b	4.11±0.55bc	70.09±1.39ab	6.59±1.08e	0.86±0.03abc	0.41±0.06bcd	0.27±0.03b	0.08±0.01bc	7.45±1.05ef	73.82±1.33ef	18.00±0.3b	10.11±2.83a
Arbequina		15.4±1.17a	4.49±0.72a	0.22±0.03bcd	0.62±0.05a	2.76±0.51d	58.89±2.47f	13.16±0.78ab	0.7±0.03e	0.37±0.05cde	0.26±0.03b	0.09±0.01b	14.75±0.75ab	63.2±2.53ab	18.16±1.16b	4.62±0.42de
Manzanila		13.67±0.46cd	2.63±0.27cde	0.37±0.07bc	0.8±0.14a	4.32±0.44ab	65.87±2.22bcd	9.44±1.61de	0.76±0.07de	0.54±0.11bc	0.25±0.04bc	0.3±0.07a	9.84±0.36de	70.58±0.65de	18.34±0.85b	7.18±0.28bc
Ezhi8		15.62±0.73a	2.48±0.27de	0.22±0.03bcd	0.32±0.08bc	2.32±0.29e	69.89±0.84a	7.58±0.96e	0.31±0.07f	0.26±0.08f	0.23±0.05b	0.21±0.06a	7.45±0.25f	73.26±0.78f	18.75±0.9b	9.85±0.27a

[1] PUFA= polyunsaturated fatty acids; MUFA= monounsaturated fatty acids; SFA= saturated fatty acids; ND: Non detected.

[2]Letters “a, b, c” indicates significant differences at a level of $p < 0.05$ by Tukey’s test between MI, within the same cultivar.

[3]Different letters indicated the significant differences at a level of $p < 0.05$ by Tukey’s test within the ten cultivar.

Table S5. Phenolic composition and oxidative stability of virgin olive oils from ten cultivars with three maturity indexes in Wudu (phenolics content expressed as mg/kg; oxidative stability expressed as h).

Cultivar	MI	Phenolic alcohols		Phenolic acids			Aldehyde	Secoiridoids					Lignans	Flavones		TPP	Oxidative stability
		Hydroxytyrosol	Tyrosol	Vanillic acid	Caffeic acid	p-coumaric acid	Vanillin	Elenolic acid	Oleacein	Oleocanthal	Oleuropein aglycone	Ligstroside aglycone	acepinoresinol	Luteolin	Apigenin		
Ulliri Bardhe	2	1.54±0.12b	2.42±0.19c	0.33±0.02c	0.53±0.02b	0.81±0.02c	0.24±0.03b	1.13±0.11	160.45±9.68a	84.38±5.83a	30.68±2.88a	9.38±0.92a	7.71±0.39ab	0.67±0.03a	1.59±0.07a	337.28±33.88a	15.44±0.27
Ulliri Bardhe	3	2.28±0.34a	3.25±0.16b	0.51±0.01b	0.64±0.03a	0.99±0.03b	0.31±0.02a	1.02±0.03	119.7±6.04b	53.49±2.72b	14.8±1.09b	6.07±0.47b	8.3±0.17a	0.45±0.01c	1.1±0.07b	289.07±5b	14.79±0.74
Ulliri Bardhe	4	0.81±0.15c	3.98±0.43a	0.83±0.05a	0.73±0.08a	1.13±0.08a	0.23±0.02b	1.29±0.26	105.17±4.98b	46.39±1.87b	14.24±1b	4.93±0.3b	7.28±0.29b	0.55±0.03b	1.59±0.05a	227.84±20.19c	12.87±0.49
Empeltre	2	1.18±0.23	3.97±0.3a	0.44±0.03a	0.44±0.04b	1.28±0.08a	0.33±0.04b	13.64±0.76a	202.82±11.29a	77.95±4.07a	34.86±4b	10.52±1.0a8	10.16±0.49b	0.23±0.02b	1.33±0.07a	401.41±12.51a	19.35±1.07
Empeltre	3	1.07±0.26	3.01±0.29b	0.57±0.06b	0.74±0.03a	0.88±0.07b	0.45±0.03a	10.25±0.49c	173.75±6.69b	64.54±5.89b	21.15±2.5b7	7.18±0.27b	12.54±1.09a	0.3±0.01a	1.84±0.12a	336.52±20.27b	18.48±0.49
Empeltre	4	1.3±0.26	2.4±0.22c	0.68±0.05c	0.51±0.02b	0.86±0.12b	0.4±0.04ab	12.08±0.26b	129.06±3.55c	46.79±3.21c	15.81±1.09a	6.06±1.13b	12.66±0.34a	0.36±0.04a	1.83±0.49a	288.34±9.71c	16.84±0.46
Koroheiki	2	2.44±0.23b	4.24±0.28a	0.6±0.03b	0.93±0.03a	1.02±0.06a	0.88±0.12a	1.11±0.11	244.89±19.11a	112.19±13.0a2	117.2±6.47a	13.36±1.39a	19.53±0.74a	1.43±0.05b	5.21±0.3a	536.67±27.08a	39.54±0.78
Koroheiki	3	2.97±0.17a	3.79±0.22b	0.86±0.06a	0.67±0.03c	1.11±0.04b	0.56±0.04b	1.14±0.03	209.8±11.51b	87.77±4.71b	91.79±6.62b	11.26±0.53b	18.79±0.46a	1.74±0.04a	4.69±0.34a	456.8±28.91b	43.69±1.12
Koroheiki	4	2.34±0.21b	5.28±0.45b	0.45±0.14b	0.76±0.02b	1.31±0.03c	0.44±0.03b	1.24±0.03	182.1±12.41b	64.96±12.18c	67.83±4.99c	8.94±0.58c	17.37±0.47b	1.27±0.15b	3.99±0.13b	349.53±14.94c	37.25±1.71
Ascolana tenera	2	3.75±0.3a	14.49±0.9a	3.46±0.35a	1.88±0.13a	0.82±0.14	1.37±0.09	15.83±0.55a	82.49±9.23a	70.03±7.35a	12.69±0.68b	8.34±0.55b	28.59±1.03a	0.13±0.01b	1.16±0.06b	262.15±24.99a	9.69±0.6
Ascolana tenera	3	3.41±0.36a	12.72±0.76ab	1.81±0.31b	1.45±0.15b	1.12±0.22	1.19±0.06	13.63±0.16b	67.29±7.6a	63.7±8.2a	9.93±0.73b	4.67±0.27b	26.11±1.04b	0.17±0.01a	1.36±0.02a	229.82±19.35a	7.61±0.59
Ascolana tenera	4	2.64±0.32b	12.1±0.96b	1.93±0.43b	1.67±0.1ab	1.41±0.56	1.27±0.2	16.82±2.55a	50.55±4.71b	36.83±2.0b2	8.58±0.62a	5.48±0.9a	23.67±1.25c	0.14±0.01b	1.23±0.04b	171.02±7.62b	7.51±0.34
Zhongshan24	2	2.01±0.34	6.27±0.45b	2.71±0.17a	1.05±0.13b	1.35±0.13a	0.9±0.18b	12.56±1.07b	204.8±9.9a	156.3±16.0a3	50.45±7.62a	7.97±0.63a	26.73±2.53a	0.14±0.02ab	1.48±0.16b	482.59±20.98a	23.16±0.55
Zhongshan24	3	1.45±0.2	7.91±0.43a	1.29±0.24b	1.19±0.19b	1.17±0.16a	1.28±0.17a	13.56±0.37ab	163.93±8.6b2	120.51±5.24b	28.8±6.25b	5.37±0.41b	22.56±2.15ab	0.17±0.02a	1.93±0.1a	398.36±25.25b	22.32±0.93
Zhongshan24	4	2.02±0.42	8.27±0.26a	1.19±0.28b	2.33±0.78a	0.89±0.08b	1.15±0.19ab	14.81±1.94a	121.4±30.95c	106.03±9.5b	19.46±2.63b	6.07±1.07b	20.66±2.29b	0.13±0.01b	1.3±0.19b	315.78±20.04c	17.35±0.62
Yuntai14	2	2.96±0.43	2.29±0.22b	2.44±0.32b	2.78±0.68a	2.45±0.18b	2.24±0.26b	0.93±0.06b	94.61±12.93a	65.72±7.14a	41.73±3.34a	8.94±1.64a	12.47±0.81b	0.11±0.01b	1.36±0.05b	313.09±12.99a	18.28±0.24
Yuntai14	3	2.14±0.36	4.31±0.48a	4.5±0.37a	1.19±0.14b	2.88±0.16a	2.73±0.32a	1.23±0.09a	72.16±4.39b	46.73±5.88b	29.11±2.94b	6.41±0.55b	15.12±1.48a	0.18±0.01a	2.16±0.17a	248.36±7.5b	17.03±0.33
Yuntai14	4	2.5±0.47	3.76±0.05a	1.68±0.2c	0.89±0.08b	1.81±0.18c	0.85±0.09c	0.74±0.05c	54.29±6.61b	38.87±1.7b	21.51±1.62c	5.35±0.38b	11.29±0.91b	0.09±0.01c	1.12±0.12b	220.28±13.4c1	15.7±0.32
Picual	2	1.2±0.34b	3.21±0.25b	2.93±0.12a	0.51±0.04c	2.58±0.17	1.15±0.13b	5.85±0.13b	103.01±8.64a	62.36±5.22a	35.23±6.85a	6.7±1.25a	1.43±0.2b	0.05±0.01b	0.95±0.07c	286.84±24.03a	25.89±0.48
Picual	3	1.58±0.35a	4.12±0.32ab	1.24±0.12c	1.87±0.21a	2.95±0.2	0.82±0.11a	4.95±0.08c	83.91±5.5b	38.84±4.07b	24.04±3.44b	4.77±0.37ab	2.49±0.53a	0.04±0.01b	1.65±0.13a	207.86±7.87b	23.32±0.37
Picual	4	1.18±0.28b	3.66±0.32a	1.6±0.1b	0.91±0.09b	2.62±0.19	1.24±0.11a	6.22±0.1a	67.7±4.62c	46.57±7.9b	19.35±3.48b	4.02±1b	1.81±0.21ab	0.06±0.01a	1.37±0.06b	192.53±22.58b	21.24±0.57
Arbequina	2	1.53±0.39ab	2.76±0.47b	2.98±0.6a1	1.83±0.11c	1.43±0.23b	1.04±0.06a	17.33±1.17a	57.45±3.67a	49.56±9.39a	14.51±1.85a	5.19±0.a55	13.67±2.09b	0.18±0.06a	0.95±0.08b	211.84±20.39c	9.41±0.34
Arbequina	3	1.17±0.35b	3.63±0.42a	1.73±0.09b	2.94±0.2b	1.67±0.16ab	0.85±0.1b	14.24±2.29ab	38.98±2.34b	25.87±3.96b	12.91±1.14ab	4.13±0.5b3	10.55±1.35a	0.1±0.01b	0.83±0.1b	155.36±5.73b	7.35±0.48
Arbequina	4	2.19±0.62a	2.42±0.19b	1.57±0.07b	3.67±0.17a	1.89±0.13a	0.55±0.08c	12.16±1.63b	28.53±1.29c	17.48±1.91b	10.64±1.54b	4.8±0.32ab	16.56±0.77a	0.15±0.01a	1.14±0.1a	122.78±12.1a	7.05±0.09

Manzanila	2	1.51±0.68b	7.24±1.04a	1.36±0.11a	1.36±0.19c	5.56±0.66a	0.75±0.07b	6.47±1.04	177.45±17.65a	126.68±19.5a4	58.32±6.5c	11.43±0.55a	14.62±0.72b	0.09±0.01c	3.11±0.55a	390.85±38.74a	17.76±1.74
Manzanila	3	2.13±0.19b	5.26±0.91ab	0.47±0.16c	2.56±0.17b	3.49±0.52b	0.43±0.12ab	7.5±0.89	127.89±3.3b	81.71±8.19b	45.08±6.06b	8.8±0.64b	17.18±1.33a	0.12±0.02b	1.32±0.14b	328.18±10.21b	15.76±0.55
Manzanila	4	3.24±0.27a	6.95±0.65b	0.75±0.09b	3.52±0.33a	4.46±0.48ab	0.56±0.09a	5.82±0.59	119.76±11.1b7	65.98±5b	30.01±2.41a	7.64±0.77b	16.62±1.09a	0.16±0.02a	1.05±0.28b	272.36±6.67c	14.58±0.51
Ezhi8	2	0.83±0.07b	3.09±0.09c	2.01±0.06b	1.43±0.12c	0.85±0.09a	0.56±0.11c	5.78±0.76	79.6±4.26a	29±1.99	47.55±6.25a	13.02±0.92a	9.58±0.73b	0.24±0.09	0.27±0.08	235.18±18.32a	13.18±0.57
Ezhi8	3	1.17±0.21a	4.72±0.39a	3.32±0.22a	1.76±0.09b	0.65±0.08ab	0.84±0.09b	6.76±0.38	54.51±5.33b	26.69±1.92	39.11±2.34b	8.69±0.83b	14.23±1.95a	0.22±0.04	0.27±0.02	199.4±10.28ab	13.02±0.7
Ezhi8	4	1.02±0.09ab	4.14±0.19b	1.74±0.11b	2.52±0.15a	0.74±0.06b	1.08±0.09a	6.15±0.71	43.9±3.35c	30.97±3.32	31.03±1.66b	6.78±0.91c	14.34±0.79a	0.33±0.03	0.31±0.03	163.35±21.99b	11.25±0.55
Cultivar		Hydroxytyrosol	Tyrosol	Vanillic acid	Caffeic acid	p-coumaric acid	Vanillin	Elenolic acid	Oleacein	Oleocanthal	Oleuropein aglycone	Ligstroside aglycone	Acetoxypinoresinol	Luteolin	Apigenin	TPP	Oxidative stability
Ulliri Bardhe		1.54±0.66de	3.22±0.72e	0.56±0.22e	0.63±0.1e	0.98±0.15c	0.26±0.04f	1.15±0.19ef	128.44±25.59b	61.42±17.81bcd	19.91±8.25cd	6.79±2.07	7.76±0.51d	0.56±0.09b	1.43±0.25bc	284.73±51.49bcd	14.37±1.25cde
Empeltre		1.18±0.24de	3.13±0.72e	0.56±0.11e	0.56±0.14e	1.01±0.22c	0.39±0.06ef	11.99±1.54bc	168.54±32.88ab	63.09±14.09bc	23.94±8.85bcd	7.92±2.16	11.79±1.37c	0.3±0.06bc	1.67±0.36b	342.09±50.79ab	18.22±1.27bc
Koroheiki		2.58±0.34ab	4.44±0.72bcd	0.64±0.2cd	0.79±0.12de	1.15±0.14c	0.63±0.21cdef	1.16±0.08def	212.26±30.09a	88.31±22.43b	92.27±22.02a	11.19±2.07	18.56±1.07b	1.48±0.22a	4.63±0.58a	447.67±84.03a	40.16±3.03a
Ascolana tenera		3.26±0.57a	13.1±1.31a	2.4±0.86ab	1.67±0.22cd	1.12±0.4c	1.28±0.14b	15.43±1.92a	66.78±15.25c	56.85±16.26bc	10.4±1.91d	6.16±1.76	26.12±2.34a	0.14±0.02d	1.25±0.1bc	220.99±43.19cde	8.27±1.16ef
Zhongshan24		1.83±0.4cd	7.48±0.98b	1.73±0.76bc	1.53±0.73c	1.13±0.23c	1.11±0.23b	13.64±1.49ab	163.38±39.84ab	127.61±24.41a	32.9±14.68bcd	6.47±1.33	23.32±3.36ab	0.15±0.02d	1.57±0.31bc	398.91±74.75ab	20.94±2.79bc
Yuntai14	mean values	2.53±0.51bc	3.45±0.94de	2.87±1.29a	1.62±0.95cd	2.38±0.49b	1.94±0.87a	0.96±0.22f	73.69±19.07c	50.44±12.84cde	30.79±9.16bcd	6.9±1.83	12.96±1.95cd	0.13±0.04d	1.55±0.48bc	260.58±42.43ade	17±1.15bcd
Picual		1.32±0.34de	3.66±0.4de7	1.92±0.78b	1.1±0.61cde	2.72±0.24b	1.07±0.22bc	5.67±0.57cd	84.87±16.3c	49.26±11.59cde	26.21±8.22bcd	5.16±1.45	1.91±0.55e	0.05±0.01d	1.32±0.32bc	229.08±46.99de	23.48±2.06b
Arbequina		1.63±0.6cde	2.94±0.63e	2.09±0.74b	2.81±0.82a	1.66±0.25b	0.81±0.23cde	14.57±2.72ab	41.65±12.88c	30.97±15.31de	12.69±2.15cd	4.71±0.62	13.59±2.91c	0.15±0.05d	0.97±0.16c	163.33±40.88e	7.94±1.15f
Manzanila		2.29±0.84bc	6.48±1.2bc	0.86±0.41cd	2.48±0.96ab	4.5±1.02a	0.58±0.16def	6.6±1.05cd	141.7±29.03b	91.46±29.37b	44.47±13.1b	9.29±1.77	16.14±1.49c	0.12±0.04d	1.83±1.02bc	330.46±55.2bc	16.03±1.68bcde
Ezhi8		1.01±0.19e	3.98±0.75cde	2.36±0.74cde	1.9±0.49bc	0.75±0.11c	0.83±0.24bcd	6.23±0.7de	59.34±16.32c	28.89±2.85e	39.23±7.94bc	9.5±2.87	12.72±2.6e	0.26±0.07cd	0.28±0.05d	199.31±34.62e	12.48±1.07def

[1] TPP= total polar phenolics.

[2]Letters “a, b, c” indicates significant differences at a level of p <0.05 by Tukey’s test between MI, within the same cultivar.

[3]Different letters indicated the significant differences at a level of p <0.05 by Tukey’s test within the ten cultivar.

Table S6. Autofit results for Orthogonal Projections to Latent Structures Discriminant Analysis (OPLS-DA) models and OPLS models, according to VOO physicochemical indicators.

Model	OPLS-DA		
	R^2Y	Q^2	Comp
Ten cultivars	0.917	0.827	3+2+0
'*Koroheiki*' vs others	0.957	0.892	1+3+0
'*Ulliri Bardhe, Empeltre, Ezhi8, Yuntai14* and *Picual*' vs others	0.932	0.813	1+3+0
'*Manzanila*' *vs* others	0.886	0.72	1+3+0
'*Arbequina*, *Ascolana tenera* and *Zhongshan24*' *vs* others	0.956	0.887	1+3+0
Maturity index[1]	0.89	0.778	1+3+0

[1] Maturity index models are OPLS. Comp = number of latent components.
